# AI-Enabled Wearable Ultrasound for Noninvasive Central Venous Pressure Monitoring

**DOI:** 10.34133/cbsystems.0653

**Published:** 2026-08-05

**Authors:** Liping Dong, Xingxuan Zhang, Meng Li, Yi Li, Jingyi Guo, Shaorong Lu, Zhenyu Peng, Chenxi Zheng, Yufei Hui, Xiaoping Shao, Feiyan Wang, Weikang Jiang, Maoyao Li, Xianshuai Wu, Xu Guo, Yingchuan Li, Yuanyi Zheng, Liping Zhang

**Affiliations:** ^1^ Department of Ultrasound, Shanghai Sixth People’s Hospital Affiliated to Shanghai Jiao Tong University School of Medicine; Shanghai Institute of Ultrasound in Medicine, Shanghai 200233, China.; ^2^ Shanghai Key Laboratory of Neuro-Ultrasound for Diagnosis and Treatment, Shanghai 200233, China.; ^3^ Department of Clinical Research Center, Shanghai Sixth People’s Hospital, Shanghai Jiao Tong University of Medicine, Shanghai 200233, China.; ^4^ Department of Biomedical Science, King’s College London, London SE1 9RT, UK.; ^5^ Department of Chemistry, University of Illinois Urbana-Champaign, Champaign, IL 61801, USA.; ^6^ Department of Emergency Medicine, Shanghai Sixth People’s Hospital, Shanghai Jiao Tong University School of Medicine, Shanghai 200233, China.; ^7^ Department of Ultrasound, The Second Affiliated Hospital of Harbin Medical University, Harbin 150001, China.; ^8^ Department of Critical Care Medicine, Shanghai Tenth People’s Hospital, Tongji University School of Medicine, Shanghai 200072, China.

## Abstract

Central venous pressure (CVP) is a key indicator of right ventricular preload, but measurement with a central venous catheter (CVC) is invasive, time-consuming, and unsuitable for bedside monitoring. We developed and clinically evaluated an artificial intelligence (AI)-enabled wearable ultrasound device for noninvasive estimation of elevated CVP by imaging the internal jugular vein (IJV) and common carotid artery (CCA) in acute and critical patients. In this prospective multi-center clinical study, 349 patients admitted to intensive care units (ICUs) at 2 tertiary hospitals underwent neck vascular imaging with the wearable ultrasound device, while CVP was measured simultaneously via CVC as the reference standard. The dual-decoder spatiotemporal attention network (DSTA-Net) segmentation model automatically quantified IJV and CCA cross-sectional areas, and the dual-modality multilayer perceptron (DM-MLP) integrated these vascular indices with basic clinical parameters to predict elevated CVP (≥8 mmHg). DSTA-Net showed excellent agreement and strong correlation with expert manual measurements while achieving superior segmentation accuracy compared with the baseline segmentation model. Meanwhile, the proposed DM-MLP improved prediction performance by 4% to 8% over conventional baseline architectures, achieving AUCs of 0.91 (internal test set) and 0.87 (external test set). These findings indicate that an AI-integrated wearable ultrasound device can provide accurate, noninvasive bedside assessment of CVP and may offer a promising alternative to invasive catheters for hemodynamic monitoring in acute and critical care settings.

## Introduction

Central venous pressure (CVP) is defined as the pressure at the junction between the right atrium and the superior vena cava. It is an important indicator of right ventricular preload and a key parameter for guiding fluid resuscitation in clinical practice. Elevated CVP is closely associated with a higher incidence of adverse events and increased mortality [1–3]. Legrand et al. [4] reported that elevated CVP was associated with a higher incidence of acute kidney injury, and other studies have linked elevated CVP to liver injury and failure [5,6]. These findings underscore the prognosticimportance of elevated CVP and highlight the need for early and accurate identification to improve patient outcomes.

Central venous catheters (CVCs), the reference standard for assessing CVP [7,8], are invasive, time-consuming, and associated with a range of procedure-related complications, including infection, thrombosis, bleeding, pneumothorax, and hemothorax [9]. These potential risks limit the broader clinical use of CVCs, particularly for routine or repeated monitoring in unstable patients. Noninvasive alternatives have been explored, including ultrasound assessment of the inferior vena cava to estimate CVP [10–12]. However, the quality of inferior vena cava imaging is frequently compromised by factors such as obesity, bowel gas, and pulmonary overinflation, which reduce its reliability in certain patient populations [13,14]. In contrast, the right internal jugular vein (IJV), which connects directly to the right atrium via a valveless pathway, has emerged as a promising surrogate for CVP assessment [15–17]. As a superficial vessel with fewer interfering factors, IJV enables more consistent and higher-quality ultrasound imaging, offering a new perspective for reliable noninvasive CVP monitoring.

Wearable ultrasound devices have recently emerged as innovative tools for bedside monitoring because of their flexibility, portability, and skin-conformal design. Several studies have shown that wearable ultrasound can be used to monitor internal structures and physiological conditions, including blood vessels, myocardium, diaphragm, gastrointestinal tract, bladder volume, and lung aeration, and can even support repeated or prolonged imaging of the left ventricle during physical activity [18–21]. Moreover, wearable ultrasound patches have demonstrated promising antitumor effects in preclinical models [22,23]. By contrast, conventional handheld ultrasound probes are relatively bulky and require continuous manual handling or mechanical stabilization, making them unsuitable for prolonged or repetitive bedside monitoring. Persistent pressure from such probes on the neck can cause discomfort and, in some acute and critical patients, may even compromise the airway. Wearable ultrasound devices offer a compact, hands-free alternative that enables repeated or prolonged monitoring with minimal patient discomfort [20,24]. Nevertheless, most current systems still rely on manual measurement, which increases clinical workload and limits scalability. Integrating wearable ultrasound with artificial intelligence (AI) has the potential to enable automatic image analysis and portable, noninvasive assessment of CVP.

To address these challenges and enhance the diagnostic utility of wearable ultrasound, we propose an integrated framework that combines a semi-supervised segmentation network with a clinical prediction model. Specifically, we developed a dual-decoder spatiotemporal attention network (DSTA-Net) for automatic segmentation of IJV and common carotid artery (CCA) from wearable neck ultrasound, and a dual-modality multilayer perceptron (DM-MLP) that integrates ultrasound-derived vascular indices with demographic and physiological features to predict elevated CVP. DSTA-Net uses a shared encoder with a dual-decoder architecture and a spatiotemporal attention mechanism to exploit both labeled and unlabeled video frames, thereby reducing the need for extensive manual annotation while maintaining accurate vascular measurements. DM-MLP employs a dual-mixing strategy to capture cross-feature dependencies and intra-feature contextual information, enabling robust and noninvasive prediction of elevated CVP. By integrating wearable ultrasound with these AI models, our study provides practical bedside solution for automated CVP assessment that can support timely hemodynamic decision-making and improve outcomes in acute and critical patients.

## Materials and Methods

### Participants and study design

This multicenter, prospective, stratified, cross-sectional clinical trial was conducted in accordance with the Declaration of Helsinki. It was approved by the institutional review board of Shanghai Sixth People’s Hospital [institutional review board number: 2023-007-(3); approval date: 2023 August 30] and Shanghai Tenth People’s Hospital (institutional review board number: SHSY-IEC-6.0/25K294/P01; approval date: 2025 October 11). The trial was registered in the Chinese Clinical Trial Registry (https://www.chictr.org.cn/; registration number: ChiCTR230007586). None of the study participants had been included in previous studies. Written informed consent was obtained from all patients or their first-degree relatives. Acute and critical patients from the intensive care unit (ICU) were enrolled at Shanghai Sixth People’s Hospital from 2023 December 1 to 2025 January 1 and at Shanghai Tenth People’s Hospital from 2025 October 11 to 2025 December 1. The inclusion criteria were as follows: (a) patients aged 18 years or older, and (b) patients who underwent CVP monitoring. The exclusion criteria, designed to eliminate confounding factors that could interfere with accurate CVP measurement, included (a) moderate to severe tricuspid regurgitation; (b) dilation of the right heart; (c) inability to lie flat due to unstable vital signs; (d) inability to visualize neck vessels; and (e) poor image quality.

### Wearable ultrasound device

We developed an ultrasound monitoring device comprising a wearable patch probe and a driving module for prolonged vascular assessment. The wearable probe integrates an ultrasound transducer as the core sensor. The transducer comprises 128 elements arranged in a linear array configuration, with a center frequency of 8.5 MHz. This frequency provides an optimal trade-off between penetration depth for imaging IJV and CCA and high spatial resolution. The transducer stack consists of a PZT-5H piezoelectric layer, dual acoustic matching layers, a backing layer, and a thin silicone encapsulation, resulting in a total thickness below 6 mm, thereby ensuring mechanical robustness while maintaining patient comfort during prolonged wear. The fabrication process of the probe is illustrated in Fig. S1.

To optimize acoustic coupling with soft tissue, a dual-layer acoustic matching structure in combination with a carefully tuned backing layer was adopted. These design features minimize interfacial reflections, broaden the operational bandwidth, and improve overall sensitivity. Performance evaluations demonstrated a fractional bandwidth of 85% ± 3% and a sensitivity of −50.0 dB ± 0.2 dB, indicating broad bandwidth characteristics and high acoustic efficiency. A detailed description of the transducer design is provided in Text S1. Detailed design parameters of the wearable patch probe are provided in Table S1 and presented graphically in Fig. S2. Additional results from continuous monitoring trials, coupling-agent dehydration studies, and thermal stability assessments are presented in Fig. S3.

The driving module follows a conventional pulse-echo ultrasound imaging architecture and integrates transmit/receive circuitry, an analog front-end signal conditioning module, analog-to-digital conversion, a main processing unit, and power management circuitry. It performs amplification, filtering, digitization, and imaging processing of the radio-frequency signals received from the probe. The processed data are transmitted to a tablet device via Wi-Fi for visualization and storage. The overall system is designed with an emphasis on miniaturization and low power consumption, enabling long-term continuous monitoring.

The device was evaluated for biocompatibility and electrical safety in accordance with GB 9706.1 and ISO 13485 standards and has received regulatory clearance as a medical device (Sichuan Medical Device Registration No. 20242060267).

### Reference standard for CVP

**Fig. 1.**
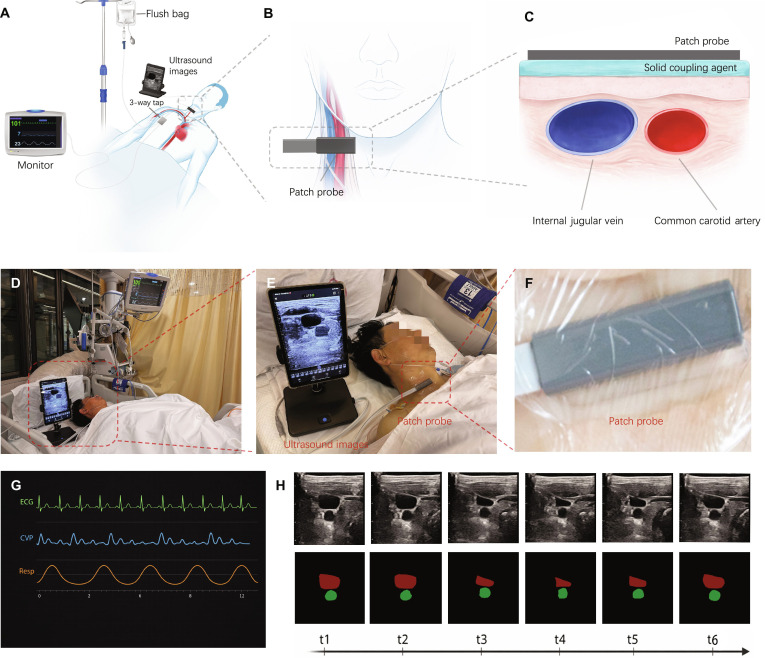
Wearable ultrasound-based monitoring of the right internal jugular vein, common carotid artery, and central venous pressure. (A to C) Schematic diagrams of the wearable ultrasound monitoring system for simultaneous assessment of the right internal jugular vein (IJV), common carotid artery (CCA), and central venous pressure (CVP). (A) Overall monitoring setup with the bedside monitor, flush bag system, 3-way tap, and ultrasound image acquisition. (B) Placement of the patch probe on the patient’s right neck. (C) Cross-sectional view of the patch probe and solid coupling agent overlying IJV and CCA. (D to F) Photographs of the wearable ultrasound monitoring process in a patient. (D) Overall bedside monitoring scene. (E) Ultrasound image acquisition and probe placement on the right neck. (F) Close-up photograph of the attached patch probe. (G) Bedside monitor displaying the CVP waveform, respiratory waveform, and electrocardiogram/HR signal. (H) Time-varying ultrasound images of the right IJV and CCA and their corresponding segmentation maps from t1 to t6.

CVP in consenting participants was assessed by a board-certified emergency physician and an experienced nurse, each of whom had performed more than 100 CVC procedures, the reference standard for CVP measurement. The catheter was inserted at the junction of the right atrium and the superior vena cava via the internal jugular, subclavian, axillary, or femoral vein and connected to a pressure transducer and bedside monitor. Correct catheter tip position was verified in all patients by chest radiography before CVP recording. The 3-way tap was positioned along the mid-axillary line at the level of the fourth intercostal space, corresponding to the midpoint of the right atrium [2,25]. Three consecutive CVP measurements were obtained, and the reference value was calculated as their mean to reduce noise and transient physiological fluctuations (Fig. 1). Because intrathoracic pressure varies with respiration, accurate timing of the measurements was essential. CVP was recorded at end-expiration during calm, regular breathing, or at the onset of expiration during spontaneous breathing to minimize respiration-induced fluctuations. When abnormal or irregular breathing patterns were observed, the measurements were repeated to ensure reliability. In accordance with septic shock treatment guidelines, acute and critical patients were classified into 2 CVP groups: a non-elevated CVP group (CVP < 8 mmHg) and an elevated CVP group (CVP ≥ 8 mmHg) [3].

### Monitoring of IJV and CCA

Before wearable ultrasound monitoring, patients were routinely positioned supine at $0^{\circ }$, without a pillow or head support, with the neck maintained in a neutral position and gently rotated to the left ($\leq 30^{\circ }$) to expose the right side of the neck. The patch probe was gently positioned at the junction of the middle and lower thirds of the neck and slightly adjusted to obtain the clearest cross-sectional ultrasound images of IJV and CCA. A solid coupling agent (Fig. S2B) was applied between the patch and the skin and secured with transparent adhesive tape. No additional pressure was applied over the patch probe, and the tape was not applied tightly so as to avoid compressing the neck vessels and causing deformation. During calm breathing, sonographers operated the wearable ultrasound device to record IJV and CCA cross-sectional videos over 3 respiratory cycles, which were saved in MP4 format. Simultaneously, waveforms and data from the CVP monitor were recorded (Fig. 1). All ultrasound measurements and initial annotations were performed by 2 sonographers with 3 and 4 years of experience, respectively, and subsequently reviewed and finalized by a senior ultrasound physician with more than 10 years of experience, and the finalized annotations were used as the ground truth for model development. The data included the following measurements: the maximum IJV area (IJV Max Area) at end-expiration, the minimum IJV area (IJV Min Area) at end-inspiration, the CCA area at end-expiration (CCA Area), the maximum IJV area/CCA area (IJV Max/CCA Area), and the change rate of IJV (IJV Ratio). These measurements were recorded for each respiratory cycle, and the mean value was then calculated. For reproducibility assessment, 20 images were randomly selected for repeated manual measurements; inter-observer agreement was evaluated based on independent measurements by the 2 sonographers, whereas intra-observer agreement was evaluated based on repeated measurements by the same sonographer on a separate occasion. All measurements were performed blinded to clinical data and previous measurement results.

### AI models

To ensure a fair evaluation and prevent data leakage, all dataset divisions were conducted at the patient level. The internal dataset from Shanghai Sixth People’s Hospital was randomly divided into training and internal test sets in an 8:2 ratio (corresponding to 218 and 54 patients, respectively). An external dataset from Shanghai Tenth People’s Hospital, comprising 77 patients, was reserved as an independent external test set to evaluate the model’s generalizability to unseen populations. All quantitative results represent the mean values derived from 3 independent runs, each employing a distinct random split of the internal dataset, whereas the external test set was kept fixed throughout.

#### Dual-decoder spatiotemporal attention network

In this study, we propose the DSTA-Net, a semi-supervised video segmentation framework that enables automatic segmentation of IJV and CCA and computes IJV Max Area, IJV Min Area, CCA Area, IJV Max/CCA Area, and IJV Ratio. In addition, we compare its segmentation performance with that of existing fully supervised models (UNet, Swin-UNet, and DeepLabV3+) and semi-supervised models (UniMatch, DWL, and AllSpark).

Dense pixel-level labeling of ultrasound videos requires substantial expert time and domain-specific expertise, making large-scale annotation infeasible. Our framework reduces this burden by requiring manual annotation of only a small number of key frames (e.g., frames at IJV Max Area and IJV Min Area) while effectively utilizing the remaining unlabeled frames. To validate the proposed framework under a realistic annotation budget, we constructed a dataset as follows. We extracted 47,800 images from neck ultrasound cine loops, of which 4,770 (approximately 10%) were manually annotated by ultrasound experts and used as labeled data. The remaining 43,030 images (approximately 90%) were treated as unlabeled frames and incorporated into the semi-supervised training framework of DSTA-Net, in which they contributed to model learning via pseudo-labeling. The core architecture uses a single shared encoder *E* that processes both labeled and unlabeled frames from the same video stream, ensuring that temporal continuity is encoded consistently across supervision boundaries. On top of this shared representation, we attach 2 task-differentiated decoders: a temporal-attention decoder *D*_1_ and a lightweight decoder *D*_2_. This design enables 2 complementary forms of perturbation: (a) input perturbation via data augmentation passed to *D*_1_; and (b) structural perturbation through the simplified *D*_2_ pathway. This synergy enforces cross-pathway consistency, allowing stable constraint learning purely via direct gradient backpropagation without relying on fragile exponential moving average (EMA) momentum updates. This formulation addresses key challenges by leveraging the intrinsic temporal structure of video while maintaining a practical, deployment-feasible model—turning every unlabeled frame into a useful training signal.

##### Decoder *D*_1_ with temporal attention

Decoder *D*_1_ is used to perform supervised learning on labeled frames and to produce refined pseudo-labels for unlabeled frames. Temporal attention is applied only on the final encoder feature $F_{t}^{K}$. Let $F_{t}^{K}$ denote the current frame feature (query) and ${\left\{F_{t+i}^{K}\right\}}_{i=-T}^{T}$ denote temporally adjacent features (keys/values). We reshape features into N=hw tokens (flattening spatial dimensions) and project them to:

$$Q=F_{t}^{K}W_{Q},\;K_{t+i}=F_{t+i}^{K}W_{K},\;V_{t+i}=F_{t+i}^{K}W_{V}$$ (1)

where $Q\in {\mathbb{R}}^{N\times d}$ and $K_{t+i},V_{t+i}\in {\mathbb{R}}^{N\times d}$. Temporal attention is computed as:

$$A_{t,i}=\text{softmax}\;\left(\frac{QK_{t+i}^{⊤}}{\sqrt{d}}\right),\;F_{t}^{\mathrm{TA}}=\sum_{i=-T}^{T}A_{t,i}V_{t+i}$$ (2)

The attended feature $F_{t}^{\text{TA}}$ is fused back to the spatial decoder path of $D_{1}$ to produce segmentation prediction:

$$y_{t}^{D_{1}}=D_{1}\left(F_{t}\;F_{t}^{\text{TA}}\right)$$ (3)

For unlabeled frames, $D_{1}$ processes augmented inputs to generate the pseudo-label ${\hat{y}}_{t}^{D_{1}}$.

##### Dual-perturbation consistency of $D_{2}$

The consistency regularization hinges upon the dual-decoder structure, where $D_{2}$ is deliberately streamlined for efficiency and robustness. Unlike $D_{1}$, which incorporates computationally intensive components like skip connections and the module (T) to leverage supervised signals and temporal context, $D_{2}$ omits these features. It relies solely on a simplified upsampling pathway from the shared encoder features to produce its prediction $y_{t}^{D_{2}}$. This architectural simplification serves 2 key purposes: It accelerates computation and, critically, it introduces a necessary structural perturbation into the consistency pipeline. This design enables a powerful dual-perturbation strategy applied to the unlabeled data $\left({\text{input}}_{t}^{\text{unlabel}}\right)$. $D_{1}$, with its richer structure, processes the augmented input $\left(\text{data}\_\mathrm{aug}\left({\text{input}}_{t}^{\text{unlabel}}\right)\right)$ to generate a refined pseudo-label ${\hat{y}}_{t}^{D_{1}}$. Concurrently, the structurally simpler $D_{2}$ processes the original, unaugmented input $\left({\text{input}}_{t}^{\text{unlabel}}\right)$ to generate $y_{t}^{D_{2}}$. By varying both the input content (via augmentation for $D_{1}$) and the network pathway (via structural omission in $D_{2}$), the model is compelled to reach consensus across distinct processing regimes, thereby enforcing a highly robust representation.

##### Training strategy and objective

The training objective is now a combination of standard supervision on labeled data and a consistency loss applied to the unlabeled data. Labeled frames are fed into $D_{1}$ for the primary supervised loss calculation: $L_{\text{sup}}=\text{CE}\left(y_{t}^{D_{1}}\;y_{t}^{\text{gt}}\right)$. For unlabeled frames, the core idea is to enforce that the segmentation prediction remains stable when the input is perturbed. Specifically, $D_{1}$ processes the augmented unlabeled input $\left(\text{data}\_\mathrm{aug}\left({\text{input}}_{t}^{\text{unlabel}}\right)\right)$ to produce the refined pseudo-label ${\hat{y}}_{t}^{D_{1}}$. Simultaneously, $D_{2}$ processes the original, unaugmented unlabeled input $\left({\text{input}}_{t}^{\text{unlabel}}\right)$ to produce $y_{t}^{D_{2}}$. The second loss term, $L_{\text{pseudo}}$, is calculated by comparing the output of $D_{2}$ with the pseudo-label generated by $D_{1}$: $L_{\text{pseudo}}=\text{CE}\left(y_{t}^{D_{2}}\;{\hat{y}}_{t}^{D_{1}}\right)$. This forces $D_{2}$ (operating on the clean input without temporal attention or skips) to agree with the more context-aware prediction from $D_{1}$ (utilizing temporal context and augmentation effects), effectively guiding the simpler decoder using the more complex one’s output on the same underlying, unlabeled content. The total loss balances the supervised signal and this consistency regularization:

$$L=L_{\text{sup}}+\lambda L_{\text{pseudo}}$$ (4)

where λ governs the influence of the consistency term.

##### Inference

Only $D_{2}$ is used during inference. All frames go through $E\to D_{2}$ to produce the final segmentation masks.

#### Dual-mixing multi-layer perceptron algorithm

**Fig. 2.**
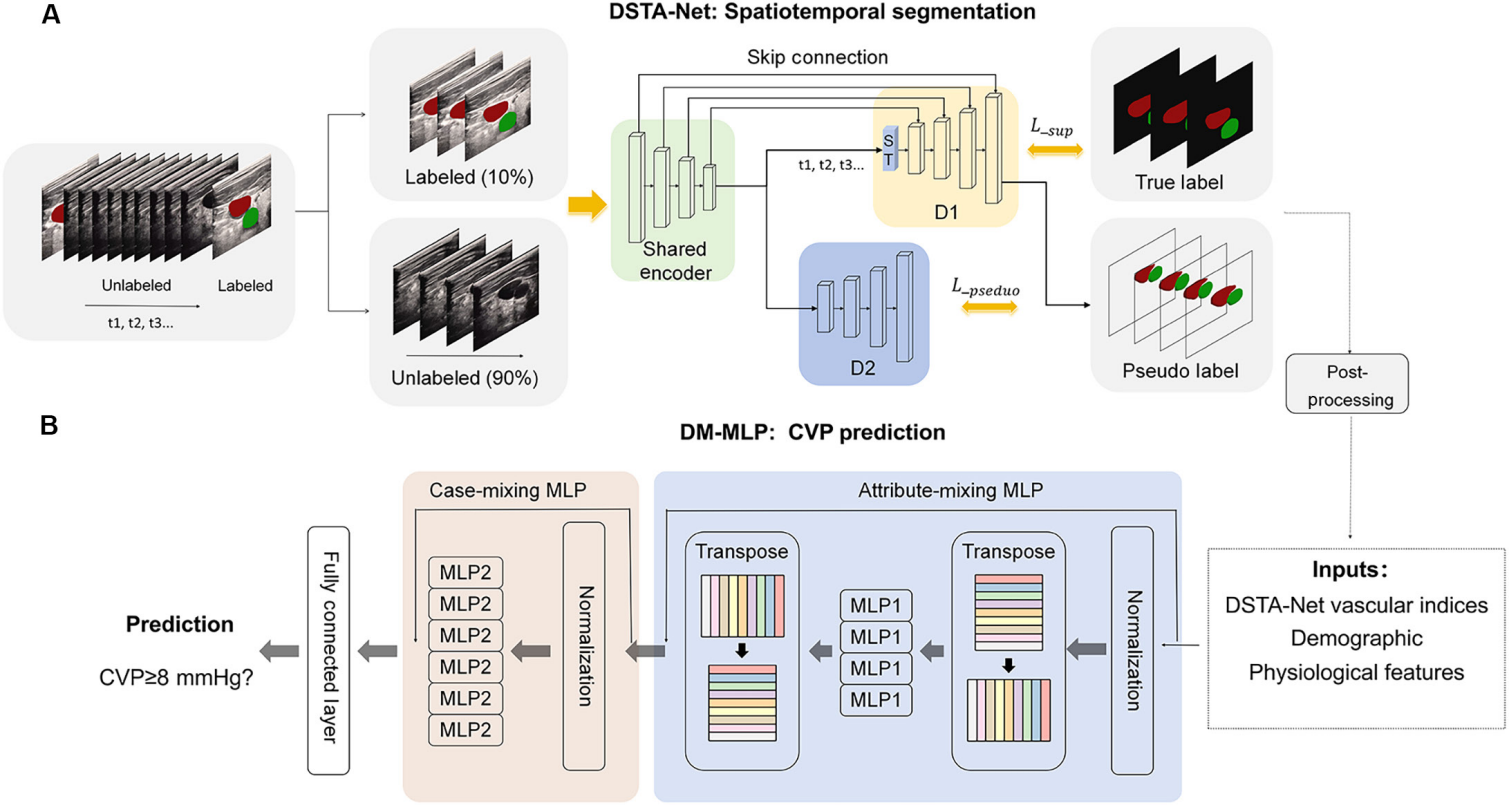
Overview of the proposed dual-decoder spatiotemporal attention network (DSTA-Net) and dual-modality multilayer perceptron (DM-MLP) framework. (A) Ultrasound videos contain a small set of manually labeled key frames (≈10%) and many unlabeled frames (≈90%) from the same temporal sequence. All frames are encoded by a shared encoder *E* and then processed by DSTA-Net (top), which comprises 2 decoders: $D_{1}$, the spatiotemporal attention decoder, and $D_{2}$, the lightweight consistency decoder. The “ST” block within $D_{1}$ denotes the spatiotemporal attention module. $D_{1}$ is supervised on labeled frames with a segmentation loss $L_{\text{sup}}$, whereas $D_{2}$ is trained on unlabeled frames via a consistency loss $L_{\text{pseudo}}$ to generate pseudo-labels. Segmented internal jugular vein (IJV) and common carotid artery (CCA) masks are post-processed to obtain vascular indices. (B) In the DM-MLP stage (bottom), 3 groups of input features are used: demographic parameters [age, height, weight, body mass index (BMI), and body surface area (BSA)], physiological features (blood pressure, HR, and respiratory rate), and DSTA-Net-derived indices (IJV Max Area, IJV Min Area, CCA Area, IJV Max/CCA Area, and IJV Ratio). These features are fed into Attribute-Mixing and Case-Mixing MLP blocks, followed by a fully connected layer that outputs the probability of elevated CVP (CVP ≥ 8 mmHg).

In this study, we propose a dual-mixing multi-layer perceptron (DM-MLP), based on the traditional MLP model, to predict elevated CVP (Fig. 2). The input to the model comprises demographic variables [gender, age, height, weight, body mass index (BMI), and body surface area (BSA)], physiological parameters [blood pressure, heart rate (HR), and respiratory rate], and ultrasound-derived indices (IJV Max Area, IJV Min Area, CCA Area, IJV Max/CCA Area, and IJV Ratio) obtained from the DSTA-Net-based segmentation of wearable ultrasound data. We formulate CVP prediction as a binary classification problem, aiming to distinguish patients with CVP ≥ 8 mmHg from those with CVP < 8 mmHg. To rigorously evaluate the effectiveness of the proposed DM-MLP, we compare its performance with 3 representative deep learning architectures: ResNet, DenseNet, and Transformer. To ensure a fair and feature-matched comparison, DM-MLP and all baseline models were trained and evaluated using exactly the same input features, preprocessing pipeline, normalization/standardization procedures, and data split settings. Therefore, model architecture was the primary experimental variable in this comparison.

The DM-MLP architecture fundamentally relies on iteratively applying 2 distinct global information-exchange mechanisms: Attribute-Mixing, which models cross-feature dependencies, and Case-Mixing, which refines the internal feature representations. These are structured within a sequence of dual-mixing blocks (DMBs). Given an input physiological feature vector $x\in {\mathbb{R}}^{D}$, the model first projects it via a linear layer $H^{\left(0\right)}=\text{Projection}\left(x\right)$. This representation then passes through *L* stacked DMBs. Each DMB incorporates residual connections and layer normalization steps around the 2 core mixing operations. The first operation is the Attribute-Mixing Layer, which performs global information exchange across all *D* features. This mechanism is crucial for learning intricate, nonlocal dependencies between distinct physiological metrics (e.g., age and BMI). To ensure superior efficiency and computational benefits over standard dense layers, we implement this layer using a low-rank bottleneck projection applied across the feature dimension *D*:

$$\begin{array}{c} Z_{\text{attr}}^{\prime }=Z_{\text{attr}}+{\text{MLP}}_{\text{attr}}\left(\text{LayerNorm}\left(Z_{\text{attr}}\right)\right) \end{array}$$ (5)

where ${\text{MLP}}_{\text{attr}}\left(Z\right)=\text{GELU}\left({\text{ZW}}_{A1}\right)W_{A2}$

Here, $W_{A1}\in {\mathbb{R}}^{D\times D_{k}}$ and $W_{A2}\in {\mathbb{R}}^{D_{k}\times D}$, where $D_{k}$ is a bottleneck dimension satisfying $D_{k}\ll D$. This low-rank structure significantly reduces the computational complexity of the global feature mixing compared to a full-rank transformation $W\in {\mathbb{R}}^{D\times D}$. Following this, an element-wise feed-forward network (FFN) refines the representation: $Z_{\mathrm{ffn}}^{\prime }=\text{FFN}\left(\text{LayerNorm}\left(Z_{\mathrm{attr}}^{\prime }\right)\right)+Z_{\mathrm{attr}}^{\prime }$. The second core operation is the Case-Mixing Layer, which performs feature-wise integration, effectively modeling contextual information within the representation of each individual physiological metric. This is executed via a standard 2-layer MLP applied across the feature dimension:

$$\begin{array}{c} H^{\left(l\right)}=\text{Case}-\text{Mixing}\left(\text{LayerNorm}\left(Z_{\mathrm{ffn}}^{\prime }\right)\right)+Z_{\mathrm{ffn}}^{\prime } \end{array}$$ (6)

where $\text{Case}-\text{Mixing}\left(Z_{\text{case}}\right)=\text{GELU}\left(Z_{\text{case}}W_{C1}\right)W_{C2}$, with $W_{C1}\in {\mathbb{R}}^{D\times D_{h}}$ and $W_{C2}\in {\mathbb{R}}^{D_{h}\times D}$. The output of the final DMB, $H^{\left(L\right)}$, is aggregated via global mean pooling and passed through a final dense layer to produce the classification logit $\hat{y}$. For the binary CVP prediction task, the model is trained to minimize the binary cross-entropy (BCE) loss augmented with $L_{2}$ regularization over all parameters Θ:

$$\begin{array}{c} L=\frac{1}{|B|}\sum_{\left(x_{i}y_{i}\right)\in B}L_{\text{BCE}}\left(y_{i}\;\sigma \left({\hat{y}}_{i}\right)\right)+\lambda ∥\Theta {∥}^{2} \end{array}$$ (7)

where the fundamental loss component $L_{\text{BCE}}$ for a sample with true label $y\in \left\{0,\;1\right\}$ and predicted probability $p=\sigma \left(\hat{y}\right)$ is defined as:

$$\begin{array}{c} L_{\text{BCE}}\left(y,\;p\right)=-\left[y\text{log}\left(p\right)+\left(1-y\right)\text{log}\left(1-p\right)\right] \end{array}$$ (8)

This overall structure ensures that the model simultaneously learns rich cross-feature correlations (via Attribute-Mixing) and deep intra-feature representations (via Case-Mixing) before making the final prognostic decision.

#### Implementation details

##### Training and deployment environment

The proposed model architecture contains approximately 225 million parameters. All training and evaluation were conducted on a cluster of 8×NVIDIA GeForceRTX4090 graphics processing units (GPUs). In the clinical deployment setting, the wearable ultrasound device uploads imaging data via the hospital intranet to the hospital server for processing. With this setup, the entire inference pipeline—including DSTA-Net segmentation and DM-MLP-based CVP prediction—achieves a processing speed of 32 frames per second (30 ms per frame), enabling fast, real-time interpretation of the uploaded imaging data solely through server-side inference, rather than on-device computation on the wearable device or tablet.

##### Optimization and hyperparameters

Both the DSTA-Net and DM-MLP components were optimized using the AdamW optimizer. The global batch size was set to 64, corresponding to 8 samples per GPU on the 8×RTX4090 configuration. For DSTA-Net, the input video clip length was fixed at 16 frames, with an initial learning rate of $2\times 10^{-4}$. The DM-MLP model was trained using a separate initial learning rate of $1\times 10^{-5}$. Weight decay was uniformly set to $1\times 10^{-4}$ for both models. Training was limited to 50 epochs and employed an early-stopping strategy with a patience of 10 epochs based on validation loss to prevent overfitting.

### Statistical analysis

Descriptive analyses were performed using R (version 3.5.0). The Shapiro–Wilk and Kolmogorov–Smirnov tests were applied to assess data normality. Normally distributed data are reported as means ± standard deviations (SDs), whereas non-normally distributed data are summarized as medians with interquartile ranges (IQRs). The Mann–Whitney *U* test was used to evaluate differences in continuous variables. Agreement and correlation between DSTA-Net-derived measurements and expert manual annotations were evaluated using Bland–Altman (B–A) and Spearman correlation analyses. For B–A analysis, percentage error was calculated as $\left(\text{DSTA}-\text{Net}-\text{Manual}\right)/\text{Manual}\times 100$, and we adopted the commonly used empirical threshold of percentage error <30% as an exploratory criterion for clinical acceptability to aid interpretation of agreement [26]. Inter- and intra-observer reliability of manual measurements in 20 images was assessed using intraclass correlation coefficients (ICCs) with 95% confidence intervals (CIs). Deep learning model development and training were performed using Python 3.9.1, PyTorch 1.10.0, and CUDA 11.3 with cuDNN 8.2. The performance of the DSTA-Net, U-Net [27], Swin-UNet [28], DeepLabV3+[29], UniMatch [30], DWL [31], and AllSpark [32] segmentation models was evaluated using the Dice score and intersection over union (IoU). Using the CVC as the reference standard, the diagnostic performance of the DM-MLP, DenseNet [33], ResNet [34], and Transformer [35] models in detecting elevated CVP was assessed using the area under the receiver operating characteristic curve (AUC–ROC), the area under the precision–recall curve (AUC–PR), and confusion matrices, with AUC values reported alongside 95% CIs. In addition to the primary analysis using 8 mmHg as the clinically defined cutoff for elevated CVP, sensitivity analyses were performed using alternative thresholds of 7 and 9 mmHg to assess robustness across clinically relevant binary definitions. Statistical differences between ROC curves were examined using DeLong’s test, and a 2-sided *P* value of <0.05 was considered statistically significant.

## Results

### Baseline patient characteristics

**Fig. 3.**
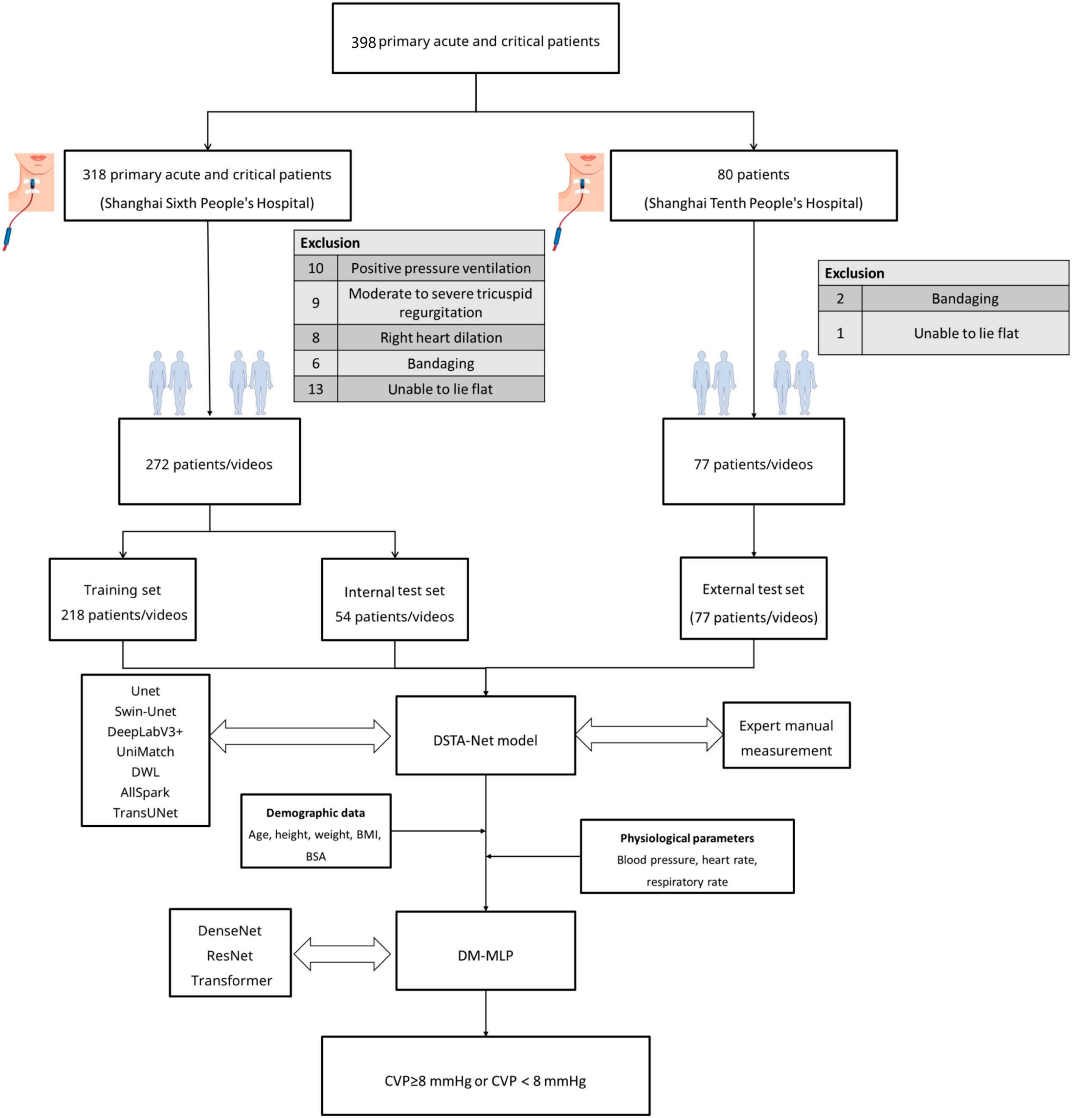
Flow of acute and critical patients receiving central venous catheterization (CVC).

A total of 318 acute and critical patients were initially enrolled at Shanghai Sixth People’s Hospital. Of these, 46 patients were excluded because of positive-pressure ventilation (*n* = 10), moderate-to-severe tricuspid regurgitation (*n* = 9), right heart dilation (*n* = 8), inaccessibility of the cervical vessels due to bandaging (*n* = 6), or inability to lie flat (*n* = 13). Ultimately, 272 patients were included in the training set (218 patients, 80%) and the internal testing set (54 patients, 20%). A total of 80 patients were initially enrolled at Shanghai Tenth People’s Hospital, with 3 patients excluded because of bandaging (*n* = 2) or inability to lie flat (*n* = 1). Seventy-seven patients were included in the external testing set. The main clinical indications for CVC among the enrolled patients included fractures, hypovolemic shock, pulmonary infection, gastrointestinal bleeding, malignant tumors, diabetic ketoacidosis, heart failure, organ rupture, cerebral hemorrhage, intestinal obstruction, acute pancreatitis, liver cirrhosis, and renal failure. The patient enrollment flowchart is shown in Fig. 3.

Within the training set, patients were divided into 2 groups based on CVP values: the elevated CVP group (*n* = 87; mean age, (58.86 ± 18.14) years [SD]; 38 women) and the non-elevated CVP group (*n* = 131; mean age, (52.21 ± 21.27) years [SD]; 77 women). The same stratification was applied to the internal and external testing sets. In the internal testing set, the CVP ≥ 8 mmHg group comprised 22 patients (mean age, (58.05 ± 16.03) years [SD]; 12 women), whereas the CVP ≥ 8 mmHg group comprised 32 patients (mean age, (52.47 ± 18.10) years [SD]; 17 women). In the external testing set, the CVP ≥ 8 mmHg group included 27 patients (mean age, (58.96 ± 17.22) years [SD]; 12 women), and the CVP ≥ 8 mmHg group included 50 patients (mean age, (50.98 ± 16.73) years [SD]; 22 women).

In the training set, both systolic blood pressure (SBP) and diastolic blood pressure (DBP) were significantly higher in the elevated CVP group than in the non-elevated CVP group. Additionally, in the training, internal testing, and external testing sets, IJV Max Area, IJV Min Area, and IJV Max/CCA Area, measured both manually and by DSTA-Net, were significantly higher in the elevated CVP group. However, IJV Ratio, derived from both manual and DSTA-Net measurements, was significantly higher in the non-elevated CVP group than in the elevated CVP group in the training and external testing sets. The baseline clinical characteristics and wearable ultrasound-derived parameters are summarized in Table 1.

**Table 1.** Baseline patient characteristics and wearable ultrasound parameters of acute and critical patients

	Training set (218 patients)			Internal test set (54 patients)			External test set (77 patients)		
	Elevated CVP(≥8 mmHg) (87 patients)	Non-elevated CVP (≥8 mmHg) (131 patients)	*P*	Elevated CVP (CVP ≥8 mmHg) (22 patients)	Non-elevated CVP (≥8 mmHg) (32 patients)	*P*	Elevated CVP (CVP ≥8 mmHg) (27 patients)	Non-elevated CVP (CVP ≥8 mmHg) (50 patients)	*P*
Indication for central venous catheterization, *n* /%									
Fracture	147 (67.43%)			25 (46.30%)			40 (51.60%)		
Hypovolemic shock	22 (10.09%)			5 (9.26%)			11 (14.29%)		
Pulmonary infection	12 (5.50%)			5 (9.26%)			4 (5.19%)		
Gastrointestinal bleeding	3 (1.38%)			5 (9.26%)			5 (6.49%)		
Malignant tumor	9 (4.13%)			3 (5.56%)			4 (5.19%)		
Diabetic ketoacidosis	6 (2.75%)			1 (1.85%)			3 (3.89%)		
Heart failure	2 (0.92%)			4 (7.41%)			4 (5.19%)		
Organ rupture	7 (3.21 %)			0			0		
Cerebral hemorrhage	3 (1.38%)			2 (3.70%)			2 (2.60%)		
Intestinal obstruction	3 (1.38%)			2 (3.70%)			2 (2.60%)		
Acute pancreatitis	3 (1.38%)			0			0		
Liver cirrhosis	1 (0.45%)			1 (1.85%)			1 (1.30%)		
Renal failure	0			1 (1.85%)			1 (1.30%)		
Age/y	58.86 ± 18.14	52.21 ± 21.27	0.018	58.05 ± 16.03	52.47 ± 18.10	0.25	58.96 ± 17.22	50.98 ± 16.73	0.052
Women/%	38 (43.68%)	77 (58.78%)	0.082	12 (54.54%)	17 (53.12%)	0.076	12 (44.44%)	22 (44%)	0.052
Height/cm	168 (160–172)	170 (160–173)	0.473	165 (160–170)	170 (163–171)	0.058	170 (160–176)	170 (158–175)	0.541
Weight/kg	65 (60–75)	67 (60–75)	0.919	67 (59–75)	65 (55–75)	0.979	66 (55–80)	67 (60–75)	0.708
BSA/m^2^	1.72 ± 0.19	1.73 ± 0.21	0.689	1.69 ± 0.18	1.71 ± 0.18	0.694	1.84 ± 0.20	1.85 ± 0.19	0.864
BMI/(kg·m^-2^)	23.94 (21.48–27.04)	22.86 (21.11–25.95)	0.386	24.78 (20.62–26.24)	23.29 (20.76–25.34)	0.271	23.44 (21.48–25.61)	24.22 (22.66–25.64)	0.281
SBP/mmHg	135 (115–148)	119 (109–131)	<0.001	120 (104–141)	119 (109–138)	0.972	124 (113–143)	128 (116–141)	0.697
DBP/mmHg	74 (64–83)	68 (62–77)	0.01	65 (57–74)	72 (60–81)	0.192	72 (60–83)	77 (69–82)	0.244
HR/bpm	89 (78–101)	89 (80–100)	0.369	90 (81–97)	98 (84–102)	0.086	94 (81–106)	90 (82–100)	0.685
Breath/(bpm·min^-1^)	20 (18–24)	20 (18–21)	0.148	20 (18–22)	20 (16–22)	0.507	22 (15–25)	21 (16–25)	0.881
CVP/mmHg	10 (8–12)	4 (3–6)	<0.001	9 (8–9)	5 (3–5.4)	<0.001	10 (8–12)	4 (3–6)	<0.001
Manual IJV Max Area /mm^2^	200.13 (164.72–270.32)	91.74 (64.27–122.38)	<0.001	210.60 (160.02–257.46)	78.41 (65.44–110.42)	<0.001	184.00 (167.40–266.44)	76.30 (36.87–118.68)	<0.001
Manual IJV Min Area/mm^2^	145.35 (96.93–189.05)	57.57 (28.15–82.54)	<0.001	138.41 (87.50–187.44)	45.11 (20.43–57.88)	<0.001	159.79 (127.28–235.22)	44.67 (24.03–79.83)	<0.001
Manual IJV Ratio	26.11 (13.25–47.95)	34.30 (18.75–57.54)	0.026	30.66 (14.00–45.43)	49.48 (18.03–72.35)	0.13	13.09 (6.14–28.00)	29.72 (19.75–46.39)	0.001
Manual CCA Area/mm^2^	59.50 (46.26–74.74)	65.31 (63.62–71.18)	0.322	68.91 (54.84–77.81)	54.35 (44.22–65.99)	0.053	50.05 (42.40–64.44)	43.89 (37.42–54.09)	0.2
Manual IJV Max/CCA Area	3.54 (2.83–4.94)	1.66 (1.18–2.28)	<0.001	3.20 (2.72–3.79)	1.55 (1.00–2.15)	<0.001	4.18 (2.43–6.28)	1.59 (0.92–2.23)	<0.001
DSTA-Net IJV Max Area /mm^2^	234.18 (184.37–292.65)	130.66 (71.40–137.17)	<0.001	236.70 (181.97–294.38)	87.95 (74.47–123.80)	<0.001	189.89 (162.33–270.92)	74.63 (42.18–122.21)	<0.001
DSTA-Net IJV Min Area /mm^2^	152.26 (101.65–199.28)	60.34 (29.15–86.74)	<0.001	144.84 (91.73–196.85)	47.61 (21.44–61.24)	<0.001	158.03 (126.15–238.69)	44.67 (26.52–80.26)	<0.001
DSTA-Net IJV Ratio	30.07 (20.17–51.85)	37.99 (22.71–58.43)	0.041	32.37 (19.83–49.15)	51.41 (22.43–75.58)	0.105	11.67 (5.52–26.84)	28.39 (18.34–48.05)	0.001
DSTA-Net CCA Area/mm^2^	64.85 (50.25–83.69)	60.02 (47.07–78.60)	0.329	74.80 (60.03–86.80)	59.37 (50.03–74.70)	0.055	47.52 (42.17–62.77)	43.20 (37.06–52.38)	0.186
DSTA-Net IJV Max/CCA Area	3.59 (3.03–5.04)	1.74 (1.15–2.31)	<0.001	3.32 (2.73–3.90)	1.59 (1.01–2.34)	<0.001	4.29 (2.57–6.58)	1.61 (0.93–2.29)	<0.001

BSA, body surface area; BMI, body mass index; SBP, systolic blood pressure; DBP, diastolic blood pressure; HR, heart rate; IJV Max Area, internal jugular vein maximal area; IJV Min Area, internal jugular vein minimal area; IJV Ratio, (IJV maximum area − IJV minimum area)/IJV maximum are; CCA Area, common carotid artery area at end-expiration; IJV Max/CCA Area, internal jugular vein maximal area/common carotid artery area at the end of expiration

To further assess potential differences that may affect model cross-center robustness, we performed a descriptive comparison between the development cohort and the external validation cohort. The prevalence of elevated CVP was broadly comparable across the training, internal testing, and external testing sets (39.9%, 40.7%, and 35.1%, respectively). Age distributions were also generally similar across centers: In all datasets, patients with elevated CVP were older on average than those with non-elevated CVP, with mean ages of approximately 58 to 59 years versus 51 to 52 years, respectively. Sex distribution showed modest cross-center differences. In the Shanghai Sixth training cohort, the non-elevated CVP group had a higher proportion of women than the elevated CVP group (58.8% versus 43.7%), whereas in the external set the female proportion was similar between groups (44.0% versus 44.4%). Regarding model inputs, the ultrasound-derived variables showed consistent directional patterns across datasets. Specifically, IJV Max Area, IJV Min Area, and IJV Max/CCA Area were higher in the elevated CVP group in the training, internal testing, and external testing sets, whereas IJV Ratio tended to be higher in the non-elevated CVP group, particularly in the training and external testing sets. Blood pressure variables (SBP and DBP) were significantly higher in the elevated CVP group in the training set, and the same overall trend was observed in the internal and external testing sets, although the differences were less pronounced, likely due to the smaller sample sizes. These descriptive comparisons are summarized in Table S2.

### Reproducibility of manual measurements

In the 20-image reproducibility analysis, manual measurements showed excellent inter- and intra-observer reliability across all parameters (Table S3). The inter-observer ICCs ranged from 0.994 to 0.999, and the intra-observer ICCs ranged from 0.994 to 0.999. All 95% CIs were close to 1.0, and all *P* values were <0.001, indicating excellent repeatability and reproducibility of the manual measurements.

### Consistency of the DSTA-Net segmentation model

**Fig. 4.**
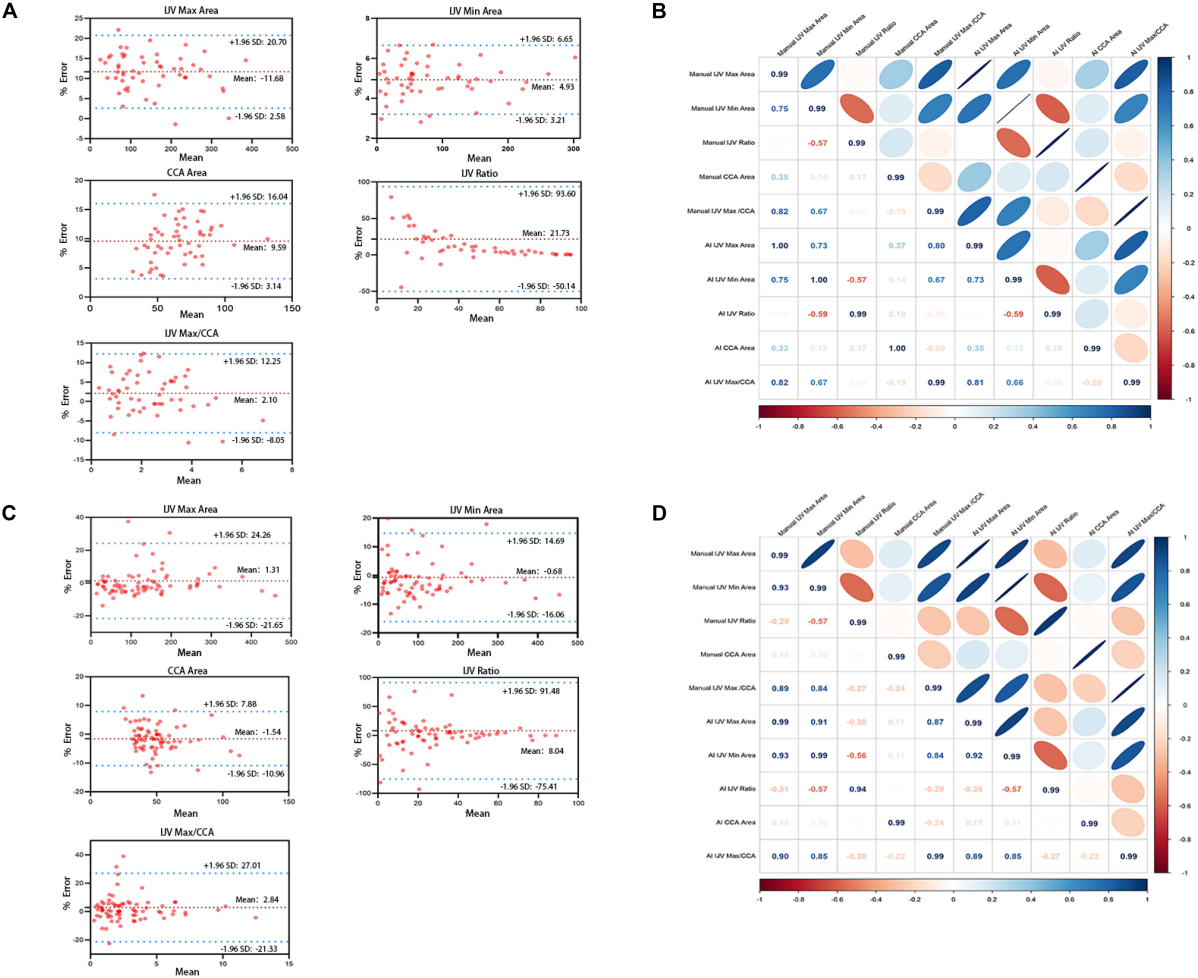
(A and C) Bland–Altman plots for the internal (A) and external (C) test sets comparing expert manual measurements with DSTA-Net-derived measurements across all ultrasound parameters. The vertical axis represents the percentage error, and the mean difference and limits of agreement (LOAs) are shown. (B and D) Spearman correlation heatmaps for the internal (B) and external (D) test sets illustrating correlations between manual and DSTA-Net measurements. The color scale ranges from red (negative correlation) to white (no correlation) to blue (positive correlation), and the numerical correlation coefficients are displayed in each cell. Strong positive correlations (*r* ≥ 0.8) indicate high concordance between the automated and expert measurements.

This study assessed the agreement between the DSTA-Net model and expert manual measurements using B–A analysis, with results expressed in terms of standardized error percentages. The findings indicated strong agreement between the model’s outputs and the expert manual measurements across multiple parameters. Notably, both the internal and external test sets exhibited minimal error variations in terms of mean differences and limits of agreement (LOAs), with the majority of parameters displaying errors below 30%, which is a threshold widely regarded as clinically acceptable [26]. Although certain parameters, such as IJV ratio measurements, displayed relatively greater error variability, the overall mean differences and LOA, when expressed as standardized error percentages, exhibited no apparent systematic bias and remained within clinically acceptable limits. These findings indicate that the DSTA-Net model provides reliable and consistent segmentation performance. Furthermore, Spearman correlation analysis demonstrated a high degree of correlation between the DSTA-Net predictions and expert manual measurements (Fig. 4 and Table S4).

### Accuracy of the DSTA-Net segmentation model

In the internal test set, DSTA-Net achieved the highest segmentation accuracy for both vessels, with Dice/IoU scores of 83.52/81.27 for IJV and 81.04/79.36 for CCA. These results represent substantial improvements over the best-performing fully supervised baseline (DeepLabv3+), with relative increases of approximately 11.9% in Dice and 11.1% in IoU for IJV, and 9.8% in Dice and 10.4% in IoU for CCA. Compared with the strongest semi-supervised competitor (UniMatch), DSTA-Net yielded relative improvements of about 4.2% in Dice and 5.2% in IoU for IJV, and 3.8% in Dice and 5.1% in IoU for CCA. This demonstrates that DSTA-Net not only establishes a new state of the art within semi-supervised methods but also outperforms fully supervised approaches under the same training conditions (Table 2).

**Table 2.** Comparison of DSTA-Net with fully supervised and semi-supervised segmentation methods on internal and external test sets. Values denote Dice and IoU scores (%) for segmentation of IJV and CCA in the internal and external test sets. DSTA-Net denotes the proposed semi-supervised method.

	Internal test set				External test set			
	Dice IJV	IoU IJV	Dice CCA	IoU CCA	Dice IJV	IoU IJV	Dice CCA	IoU CCA
Fully supervised segmentation								
UNet	72.95	70.88	72.44	70.83	66.74	65.36	65.77	65.1
Swim-UNet	71.04	68.23	70.63	67.90	65.93	61.47	64.53	62.81
DeepLabv3+	74.65	73.13	73.82	71.91	67.44	67.14	68.56	65.13
Semi-supervised segmentation								
UniMatch	80.18	77.28	78.05	75.52	73.85	70.36	70.48	70.09
DWL	79.82	77.91	76.26	74.16	73.2	70.49	69.98	67.74
AllSpark	76.33	73.81	74.51	71.99	70.69	67.09	68.82	65.51
DSTA-Net	83.52	81.27	81.04	79.36	75.83	74.77	74.28	73.29

IJV, internal jugular vein; CCA, common carotid artery; DSTA-Net, dual-decoder spatiotemporal attention network

To evaluate cross-center robustness, all models were further assessed in the external test set. DSTA-Net again achieved the best overall performance, with Dice scores of 75.83 (IJV) and 74.28 (CCA) and corresponding IoU scores of 74.77 and 73.29. These results outperformed the best-performing fully supervised (DeepLabv3+) and semi-supervised (UniMatch) by approximately 8% to 10% in both Dice and IoU metrics for each vessel. These findings suggest that, under similar acquisition conditions across the 2 hospitals, DSTA-Net learns transferable feature representations and maintains stable performance on this external validation set.

#### Segmentation results visualization

**Fig. 5.**
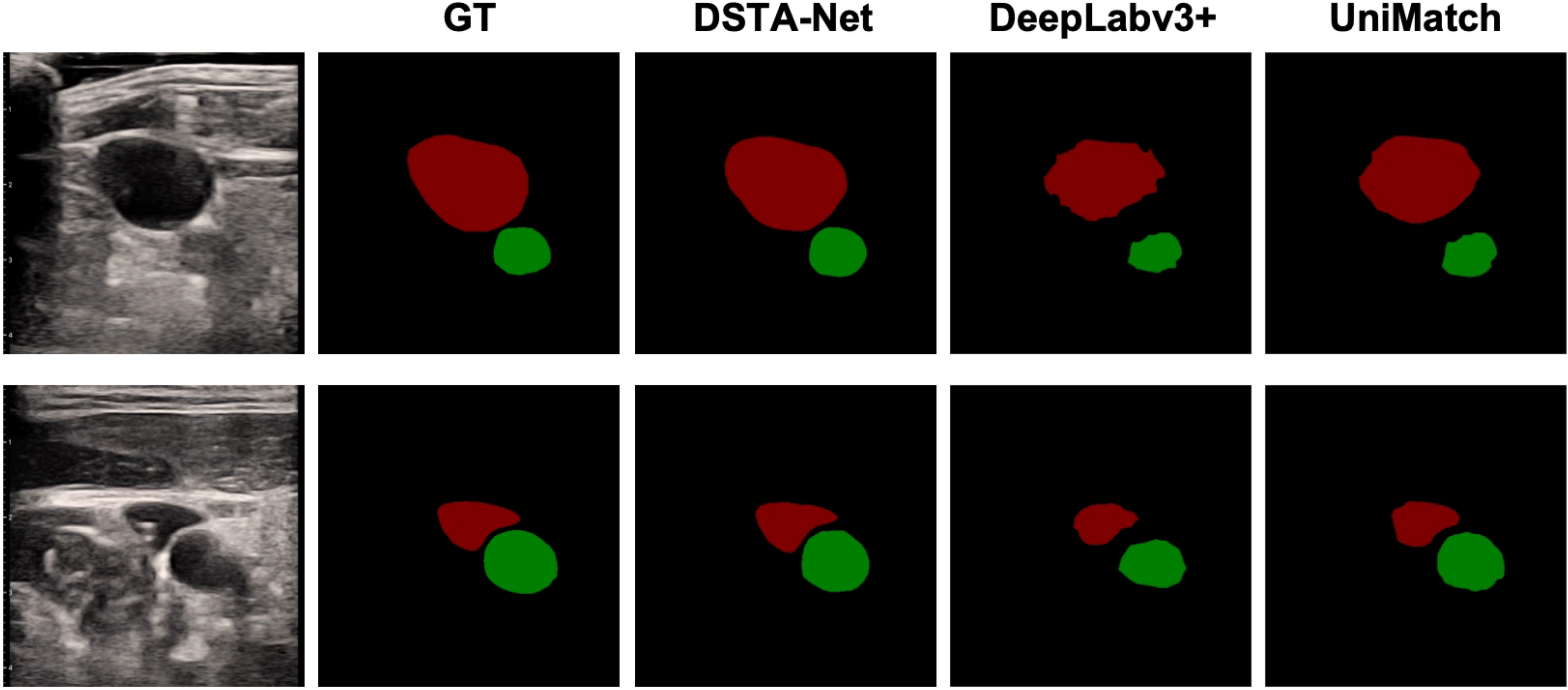
Visual comparison of segmentation results for representative samples. From left to right, each row shows the original image, ground truth (GT), our DSTA-Net, the fully supervised baseline DeepLabv3+, and the high-performing semi-supervised baseline UniMatch.

We present visual comparisons of segmentation outputs for representative images, highlighting the performance of the proposed DSTA-Net model against the 2 strongest baseline approaches: the fully supervised DeepLabv3+ and the leading semi-supervised method UniMatch. To facilitate direct evaluation, the results for each representative sample are arranged in a consistent sequence: the original image, followed by the ground truth (GT) annotation, then the predictions from DSTA-Net, DeepLabv3+, and UniMatch (Fig. 5).

### Evaluation of DM-MLP model

**Fig. 6.**
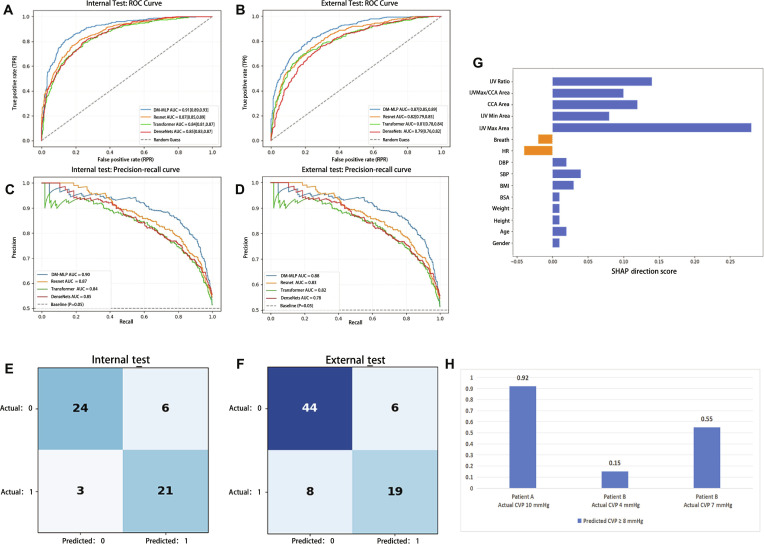
Comprehensive performance evaluation and interpretability analysis of the DM-MLP model compared with state-of-the-art baselines. (A and B) Receiver operating characteristic (ROC) curves in the internal (A) and external (B) test sets . The DM-MLP model (blue line) achieved the highest area under the curve (AUC) in both datasets compared with ResNet, Transformer, and DenseNet, indicating superior sensitivity and specificity. (C and D) Precision–recall (PR) curves for the internal (C) and external (D) test sets. The DM-MLP model achieved the largest area under the PR curve, representing an optimal trade-off between precision and recall relative to other deep learning models. (E and F) Confusion matrices for the internal (E) and external (F) test sets. DM-MLP shows balanced classification performance with low false-negative rates across both cohorts. (G) SHapley Additive exPlanations (SHAP) direction score plot for the DM-MLP model. Features are ranked by their contribution to the model predictions. Blue bars indicate a positive association with the outcome, while orange bars (e.g., HR and breath) suggest a negative association. Anatomical features such as “IJV Max Area” and “CCA Area” are identified as key predictors. (H) Representative patient-level predictions. DM-MLP assigns higher predicted probabilities to cases with elevated CVP (e.g., patient A) and lower probabilities to non-elevated or normal cases (patients B and C), illustrating its clinical discriminative capability.

All models were trained and evaluated using clinical parameters and vascular indices automatically derived from DSTA-Net segmentation outputs. Across the both internal and external sets, DM-MLP consistently outperformed the conventional deep learning architectures. In the internal test set, DM-MLP achieved an AUC-ROC of 0.91 (95% CI, 0.89 to 0.93), outperforming ResNet (AUC = 0.87; 95% CI, 0.85 to 0.89), DenseNet (AUC = 0.85, 95% CI 0.83 to 0.87), and Transformer (AUC = 0.84, 95% CI 0.81 to 0.87), corresponding to absolute AUC gains of 0.04, 0.06, and 0.07, respectively. Furthermore, we assessed the cross-center robustness of all models on the external set. DM-MLP achieved an AUC of 0.87 (95% CI, 0.85 to 0.89), exceeding ResNet (AUC = 0.82; 95% CI, 0.79 to 0.85), Transformer (AUC = 0.81; 95% CI, 0.78 to 0.84), and DenseNet (AUC = 0.79; 95% CI, 0.76 to 0.82), with absolute AUC improvements of 0.05, 0.06, and 0.08, respectively. The PR-AUC of the DM-MLP model was 0.90 (internal) and 0.88 (external), respectively, outperforming DenseNet, ResNet, and Transformer (Fig. 6A to D). The corresponding ROC and PR curves are summarized in Tables S5 and S7. As shown in the confusion matrices (Fig. 6E and F), DM-MLP achieved accuracies of 0.83 and 0.82 in the internal and external test sets, respectively. The model demonstrated balanced sensitivity and specificity across both datasets, with a low false-negative rate in the internal cohort and only a modest increase under cross-center testing. These results indicate stable classification performance and consistent cross-center performance across the 2 cohorts under comparable conditions when predicting elevated CVP. To further assess the robustness of the selected binary threshold, sensitivity analyses were performed using alternative CVP cutoffs of 7 and 9 mmHg. At the 7 mmHg threshold, DM-MLP achieved the highest ROC-AUC values of 0.89 and 0.87 in the internal and external test sets, respectively. At the 9 mmHg threshold, DM-MLP also remained the best-performing model, with ROC-AUC values of 0.91 and 0.86 in the internal and external test sets, respectively. These results further support the robustness of the proposed method across clinically relevant thresholds.

To evaluate the interpretability of DM-MLP in predicting elevated CVP, we employed SHapley Additive exPlanations (SHAP) analysis to quantify the contribution of each input feature to the model predictions (Fig. 6G). The results showed that the 5 wearable ultrasound-derived vascular indices were the primary contributors of the model’s predictions, among which IJV Max Area had the highest mean absolute SHAP value, followed by IJV Ratio, IJV Max/CCA Area, CCA Area, and IJV Min Area, all exerting positive effects. In contrast, conventional clinical parameters such as SBP, DBP, BMI, and BSA were substantially less important and only provided limited adjustment for borderline cases. Notably, HR and breath rate exhibited overall negative SHAP values. Representative patient-level visualizations further illustrate the discrimination between elevated and non-elevated CVP cases and the potential application of DM-MLP for bedside risk stratification and clinical decision support (Fig. 6H).

### Ablation studies

#### Attention module in DSTA-Net

**Fig. 7.**
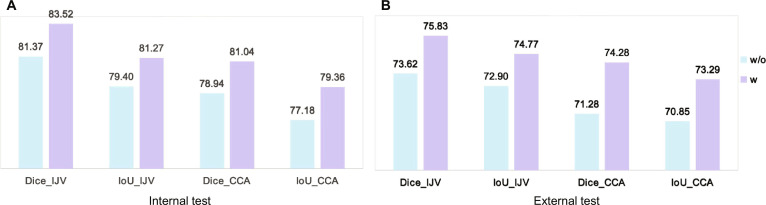
(A) Internal test performance and (B) external test performance of DSTA-Net with attention (w) and without attention (w/o). Bars indicate Dice and IoU scores for the internal jugular vein (Dice_IJV, IoU_IJV) and the common carotid artery (Dice_CCA, IoU_CCA). Across both datasets, inclusion of the attention module consistently improves Dice and IoU for both vessels, with relatively larger gains observed in the external test set.

An ablation study was performed to evaluate the specific contribution of the proposed attention mechanism in DSTA-Net. We compared the complete model equipped with attention (“w”) against a variant lacking this module (“w/o”) across both the internal and external clinical test sets. As illustrated in Fig. 7, removal of the attention module consistently resulted in reduced segmentation performance, underscoring that the model encountered difficulties in capturing vessel motion dynamics without the ability to model long-range contextual dependencies. By contrast, inclusion of the attention mechanism markedly enhanced segmentation accuracy and improved the overall quality of vascular boundary delineation and representation, highlighting its essential role in achieving stable feature alignment across successive frames. Notably, this performance benefit was evident not only on the internal dataset but also—and even more distinctly—on the external dataset, where the attention module proved particularly valuable in mitigating the effects of larger domain shifts.

#### Mixing module in dual-mixing MLP

An ablation study conducted on the internal test set examined the individual and combined contributions of the 2 primary components in DM-MLP: Attribute-Mixing (Att-Mix) and Case-Mixing (Case-Mix) (Table 3). The results revealed that Case-Mix alone produced a more substantial performance uplift (accuracy of 0.796) compared to Att-Mix alone (accuracy of 0.778), indicating that the instance-level variations introduced by Case-Mixing offer stronger regularization in data-constrained clinical scenarios. When both mechanisms were combined (Att-Mix ✓, Case-Mix ✓), the model attained the highest accuracy of 0.833, representing a notable increase from the 0.796 achieved with Case-Mix alone. This improvement was consistently reflected across precision, recall, and specificity metrics. These observations suggest that the 2 mixing strategies function in a complementary manner, jointly capturing complex feature interactions and rich instance-specific representations rather than duplicating effects. A comparable pattern emerged in the external test set, where the full model again yielded the best performance (accuracy of 0.818), albeit with somewhat reduced gains attributable to domain shift. Overall, these findings underscore the value of the dual-mixing design in bolstering feature robustness and cross-center consistency across the 2 hospitals in this study.

**Table 3.** Ablation study of DM-MLP on internal and external test sets. “Att-Mix” and “Case-Mix” denote array-level and case-level data mixing, respectively. ✓ and × indicate that the corresponding component is enabled or disabled.

Att-Mix	Case-Mix	Acc	Prec	Rec	Spe
Internal test					
×	✓	0.796	0.852	0.767	0.833
✓	×	0.778	0.846	0.733	0.833
✓	✓	0.833	0.889	0.800	0.875
External test					
×	✓	0.785	0.824	0.884	0.690
✓	×	0.773	0.851	0.800	0.720
✓	✓	0.818	0.846	0.880	0.704

Acc, accuracy; Prec, precision; Rec, recall; Spe, specificity

## Discussion

In this study, we employed a wearable ultrasound device to monitor the IJV and CCA at the bedside in acute and critical patients and developed an integrated AI framework comprising the DSTA-Net segmentation model and the DM-MLP prediction model. DSTA-Net automatically segmented IJV and CCA from neck ultrasound cine loops, and DM-MLP combined ultrasound-derived vascular indices with clinical parameters to identify elevated CVP. Taken together, the proposed wearable ultrasound–AI framework offers a promising avenue for timely hemodynamic evaluation in acute and critical care settings. It may offer a safer, noninvasive option for patients in whom invasive catheterization is contraindicated or technically difficult. By enabling early bedside screening for elevated CVP, the proposed framework may facilitate timely intervention and more precise hemodynamic management, thereby potentially improving patient outcomes while reducing healthcare burden.

Although CVP is a continuous physiological parameter, 8 mmHg was used as the primary cutoff in this study because it is a commonly adopted clinical threshold for elevated CVP [1,36]. Therefore, the model should be regarded as a screening tool for elevated CVP rather than a substitute for continuous invasive monitoring. We further assessed the stability of this model performance by applying alternative cutoffs of 7 and 9 mmHg. In both the internal and external test sets, DM-MLP remained the best-performing model. This suggests that the advantage of the proposed method is maintained across clinically relevant threshold definitions. Even so, dichotomization cannot fully capture the hemodynamic variation, and future work should move toward direct prediction of continuous CVP.

Our findings demonstrated that the IJV Max Area, IJV Min Area, and IJV Max/CCA Area were significantly higher in patients with elevated CVP. These observations are in agreement with Bayraktar and Kaçmaz [13], who reported a significant correlation between CVP and IJV diameter and supported the use of IJV measurements as a noninvasive surrogate for CVP. Similar associations between IJV filling and CVP have been reported in several independent studies [37] [38]. Taken together, these findings support the use of IJV as a noninvasive surrogate for CVP and underscore the need for practical tools that can reliably capture jugular venous congestion at the bedside.

Wearable ultrasound technology offers a promising solution to this need by enabling repeated, operator-independent assessment of neck vasculature in acute and critical patients. To date, however, relatively few studies have deployed wearable ultrasound in prospective clinical cohorts to rigorously evaluate its clinical utility [18–21]. In our multi-center study, the proposed wearable ultrasound patch probe confers several engineering advantages over conventional handheld probes and existing wearable designs [20,24]. Its ultrathin 128-element linear-array transducer, incorporating optimized dual-layer acoustic matching and a tailored backing structure, enables high-quality imaging in a compact, bedside-compatible form factor. In a supplementary 24-h feasibility experiment, the device maintained stable image quality during continuous wear, suggesting that it can support prolonged vascular monitoring in clinical settings. The use of solid hydrogels mitigates the dehydration and instability issues associated with traditional liquid coupling media, whereas the thermally stable silicone encapsulation maintains the surface temperature within a comfortable range for the skin, an important consideration for patient safety and tolerability [19,20]. Together, these hardware characteristics provide reliable input data for AI models and underscore the potential of wearable ultrasound for use in emergency and intensive care environments.

Existing semi-supervised segmentation methods are predominantly designed for static images, often overlooking the temporal continuity inherent in video data and relying on complex and sometimes unstable EMA teacher mechanisms [39,40], which can limit their generalization and practical deployability. In contrast, DSTA-Net leverages a shared encoder with a dual-decoder architecture to jointly model cross-frame dynamics and replaces the EMA teacher with direct gradient backpropagation. This design not only reduces model complexity but also transforms unlabeled frames into effective training signals. In our experiments, DSTA-Net achieved substantially higher Dice and IoU scores for IJV and CCA segmentation than the best fully supervised models (e.g., DeepLabV3+ [29]) and state-of-the-art semi-supervised methods (e.g., UniMatch [30]) on both internal and external test sets. In particular, its IJV Dice score exceeded the supervised top baseline by more than 10% in the internal and external test sets, indicating strong robustness in cross-center segmentation consistency, boundary localization, and feature representation. Beyond aggregate segmentation metrics, the vascular indices derived from DSTA-Net showed strong agreement and correlation with expert manual measurements. Notably, the IJV ratio showed wider LOAs than other ultrasound-derived parameters, likely due to its high sensitivity to respiratory-induced intrathoracic pressure changes. In acute and critical patients, heterogeneous respiratory patterns, including spontaneous breathing and mechanical ventilation, may further increase variability in venous caliber across respiratory phases. Despite this variability, DSTA-Net not only outperformed well-established semi-supervised approaches but also outperformed fully supervised architectures under the conditions of our study. Furthermore, it achieves segmentation accuracy on par with expert manual delineations while providing a more scalable and clinically viable solution for processing extensive collections of unlabeled ultrasound video data.

In this study, we developed the DM-MLP model to address limitations of existing deep learning approaches for CVP prediction by enabling efficient global feature interaction across heterogeneous demographic, physiological, and ultrasound-derived vascular indices. Through its dual-mixing design, DM-MLP captures complex nonlinear relationships that are difficult for generic ResNet-, Transformer-, or DenseNet-based architectures [33,35,41] to exploit effectively in tabular clinical data. Compared with the baseline models, DM-MLP consistently showed the best predictive performance. Its AUC-ROC reached 0.91 in the internal test cohort, representing absolute gains of 0.04 to 0.07, and 0.87 in the external cohort, with gains of 0.05 to 0.08. These findings support the robustness and clinical relevance of DM-MLP, including across the 2 hospitals in this study. From a cross-center robustness perspective, domain shift is most likely to affect the ultrasound-derived inputs, because these features depend on acquisition and labeling practices that may vary across centers. Among them, phase-dependent venous features, such as IJV Ratio, IJV Min Area, and IJV Max Area, are expected to be the most sensitive, as they are more influenced by respiratory pattern and phase selection. By contrast, demographic variables and physiological parameters are likely to be less sensitive to such variation. Therefore, the proposed model is expected to perform most consistently on cohorts acquired under conditions similar to those in the present study, namely, spontaneously breathing patients in the supine position examined using a standardized IJV/CCA ultrasound protocol. Given that validation was limited to 2 hospitals with a relatively modest sample size, its performance under different ultrasound acquisition protocols, hardware settings, or in mechanically ventilated populations requires further validation. Furthermore, the integration of SHAP analysis and representative patient-level visualizations bolsters the model’s interpretability by offering transparent, interpretable insights into its predictive reasoning. Notably, SHAP values reflect each feature’s contribution to model predictions rather than causal physiological effects.

Our research still has certain limitations. First, although this study was conducted across multiplecenters and both DSTA-Net and DM-MLP demonstrated high accuracy, the overall clinical sample size remained modest, and formal *K*-fold cross-validation was not performed. In addition, unmeasured cross-center factors, such as center-specific case mix or operator-related variation, may also have contributed to performance differences between the development and external validation cohorts. Future studies will expand the multicenter cohort to include patients with greater heterogeneity in age, comorbidities, and disease severity, incorporate cross-validation on larger and more diverse datasets, explore direct prediction of continuous CVP values, and more rigorously assess model stability and cross-center robustness. Second, the study primarily focuses on diagnostic performance rather than downstream clinical outcomes. While cross-center robustness was observed, this should be interpreted cautiously given the relatively similar acquisition conditions of the included datasets. Further validation in larger, more diverse multi-center cohorts is warranted to confirm generalizability. Prospective interventional studies are also needed to assess whether AI-guided, noninvasive CVP monitoring can improve fluid and vasopressor management and support prolonged bedside monitoring in routine clinical workflows. Third, our semi-supervised strategy assumes that frames near the most discriminative vascular states (peak dilation and peak constriction) can be treated as labeled data, while the remaining frames are considered unlabeled, leading to heterogeneous labeled-to-unlabeled ratios across patients (approximately 1:9). Future research will systematically examine how different supervision ratios affect semi-supervised segmentation performance and explore adaptive sampling or pseudo-labeling schemes tailored to nonuniform temporal distributions. Finally, although SHAP analysis was used to improve the interpretability of the DM-MLP component, the internal decision-making process of the semi-supervised segmentation backbone DSTA-Net remains relatively opaque, which may limit clinical adoption. As a future direction, we plan to integrate post hoc interpretability techniques, such as Grad-CAM, and perform quantitative analyses of learned attention patterns to clarify how semi-supervised dynamics influence feature alignment during training.

In this multi-center prospective study, we demonstrate that a neck-worn wearable ultrasound patch, integrated with an AI analysis framework, enables noninvasive identification of evelated CVP in acute and critical patients. The proposed system combines an ultra-thin array patch for reliable, long-term imaging of IJV and CCA, a DSTA-Net segmentation model that capitalizes on unlabeled cine-loop data to enhance vessel delineation, and a DM-MLP model that fuses ultrasound vascular indices with demographic and physiological parameters to deliver robust and interpretable CVP predictions. Collectively, this wearable ultrasound framework facilitates scalable, bedside hemodynamic monitoring and lays the groundwork for future interventional trials evaluating its impact on fluid management and clinical outcomes

## Data Availability

Data generated or analyzed during the study are available from the corresponding authors by request.

## References

[1] Pesenti A, Slobod D, Magder S. The forgotten relevance of central venous pressure monitoring. Intensive Care Med. 2023;49(7):868–870. 37294343 10.1007/s00134-023-07101-z

[2] Hamzaoui O, Teboul JL. Central venous pressure (CVP). Intensive Care Med. 2022;48(10):1498–1500. 35953675 10.1007/s00134-022-06835-6

[3] Hill B, Smith C. Central venous pressure monitoring in critical care settings. Br J Nurs. 2021;30(4):230–236. 33641398 10.12968/bjon.2021.30.4.230

[4] Legrand M, Dupuis C, Simon C, Gayat E, Mateo J, Lukaszewicz AC, Payen D. Association between systemic hemodynamics and septic acute kidney injury in critically ill patients: A retrospective observational study. Crit Care. 2013;17(6):R278. 24289206 10.1186/cc13133 PMC4056656

[5] Sherlock S. The liver in heart failure relation of anatomical, functional, and circulatory changes. Br Heart J. 1951;13(3):273–293. 14848381 10.1136/hrt.13.3.273 PMC479419

[6] Brienza N, Revelly JP, Ayuse T, Robotham JL. Effects of PEEP on liver arterial and venous blood flows. Am J Respir Crit Care Med. 1995;152(2, 504):510. 10.1164/ajrccm.152.2.7633699 7633699

[7] Cole R. Does central venous pressure predict fluid responsiveness? Chest. 2008;134(6):1351–1352. 19059974 10.1378/chest.08-1846

[8] Huang CY, Güiza F, Wouters P, Mebis L, Carra G, Gunst J, Meersseman P, Casaer M, van den Berghe G, de Vlieger G, et al. Development and validation of the creatinine clearance predictor machine learning models in critically ill adults. Crit Care. 2023;27(1):272. 37415234 10.1186/s13054-023-04553-z PMC10327364

[9] McGee DC, Gould MK. Preventing complications of central venous catheterization. N Engl J Med. 2003;348(12):1123–1133. 12646670 10.1056/NEJMra011883

[10] Ruge M, Marhefka GD. IVC measurement for the noninvasive evaluation of central venous pressure. J Echocardiogr. 2022;20(3):133–143. 35362870 10.1007/s12574-022-00569-6

[11] Huguet R, Fard D, d’Humieres T, Brault-Meslin O, Faivre L, Nahory L, Dubois-Randé JL, Ternacle J, Oliver L, Lim P. Three-dimensional inferior vena cava for assessing central venous pressure in patients with cardiogenic shock. J Am Soc Echocardiogr. 2018;31(9):1034–1043. 29908724 10.1016/j.echo.2018.04.003

[12] Seo Y, Iida N, Yamamoto M, Machino-Ohtsuka T, Ishizu T, Aonuma K. Estimation of central venous pressure using the ratio of short to long diameter from cross-sectional images of the inferior vena cava. J Am Soc Echocardiogr. 2017;30:461–467. 28065586 10.1016/j.echo.2016.12.002

[13] Bayraktar M, Kaçmaz M. Correlation of internal jugular vein, common carotid artery, femoral artery and femoral vein diameters with central venous pressure. Medicine. 2022;101(43): Article e31207. 36316929 10.1097/MD.0000000000031207 PMC9622599

[14] Sekiguchi H, Seaburg LA, Suzuki J, Astorne WJ, Patel AS, Keller AS, Gajic O, Kashani KB. Central venous pressure and ultrasonographic measurement correlation and their associations with intradialytic adverse events in hospitalized patients: A prospective observational study. J Crit Care. 2018;44:168–174. 29132056 10.1016/j.jcrc.2017.10.039

[15] Wang L, Harrison J, Dranow E, Aliyev N, Khor L. Accuracy of ultrasound jugular venous pressure height in predicting central venous congestion. Ann Intern Med. 2022;175(3):344–351. 34958600 10.7326/M21-2781

[16] Simon MA, Kliner DE, Girod JP, Moguillansky D, Villanueva FS, Pacella JJ. Detection of elevated right atrial pressure using a simple bedside ultrasound measure. Am Heart J. 2010;159:421–427. 20211304 10.1016/j.ahj.2010.01.004

[17] Simon MA, Schnatz RG, Romeo JD, Pacella JJ. Bedside ultrasound assessment of jugular venous compliance as a potential point-of-care method to predict acute decompensated heart failure 30-day readmission. J Am Heart Assoc. 2018;7(15): Article e008184. 30371245 10.1161/JAHA.117.008184 PMC6201476

[18] Kenny J-ÉS, Munding CE, Eibl AM, Eibl JK. Wearable ultrasound and provocative hemodynamics: A view of the future. Crit Care. 2022;26(1):329. 36284332 10.1186/s13054-022-04206-7 PMC9597974

[19] Hu H, Hu C, Guo W, Zhu B, Wang S. Wearable ultrasound devices: An emerging era for biomedicine and clinical translation. Ultrasonics. 2024;142: Article 107401. 39004039 10.1016/j.ultras.2024.107401

[20] Hu H, Huang H. A wearable ultrasound patch for continuous heart imaging. Nature. 2023;613:667–675. 36697727 10.1038/d41586-022-04535-1

[21] Burgess MT, Gluskin J, Pinker K. From bedside to portable and wearable: Development of a conformable ultrasound patch for deep breast tissue imaging. Mol Oncol. 2023;17:1947–1949. 37766480 10.1002/1878-0261.13531 PMC10552885

[22] Xue H, Jin J, Huang X, Tan Z, Zeng Y, Lu G, Hu X, Chen K, Su Y, Hu X, et al. Wearable flexible ultrasound microneedle patch for cancer immunotherapy. Nat Commun. 2025;16:2650. 40102412 10.1038/s41467-025-58075-z PMC11920228

[23] Zou F, Luo Y, Zhuang W, Xu T. A fully integrated conformal wearable ultrasound patch for continuous sonodynamic therapy. Adv Mater. 2024;36:2409528. 10.1002/adma.202409528 39104292

[24] Jo HG, Park BH, Joung DY, Jo JK, Hoh JK, Choi WY, Park KK. Forward-looking ultrasound wearable scanner system for estimation of urinary bladder volume. Sensors. 2021;21:5445. 34450887 10.3390/s21165445 PMC8400094

[25] Roger C, Muller L, Riou B, Molinari N, Louart B, Kerbrat H, Teboul JL, Lefrant JY. Comparison of different techniques of central venous pressure measurement in mechanically ventilated critically ill patients. Br J Anaesth. 2017;118:223–231. 28100526 10.1093/bja/aew386

[26] Peyton PJ, Chong SW. Minimally invasive measurement of cardiac output during surgery and critical care: A meta-analysis of accuracy and precision. Anesthesiology. 2010;113:1220–1235. 20881596 10.1097/ALN.0b013e3181ee3130

[27] Ronneberger O, Fischer P, Brox T. U-net: Convolutional networks for biomedical image segmentation. In: International Conference on Medical image computing and computer-assisted intervention. Cham (Switzerland): Springer; 2015. p. 234–241.

[28] Cao H, Wang Y, Chen J, Jiang D, Zhang X, Tian Q, Wang M. Swin-unet: Unet-like pure transformer for medical image segmentation. In: *European Conference on Computer Vision*. Cham (Switzerland): Springer; 2022. p. 205–218.

[29] Chen LC, Papandreou G, Schroff F, Adam H. Rethinking atrous convolution for semantic image segmentation. arXiv. 2017. 10.48550/arXiv.1706.05587

[30] Xu H, Zhang J, Cai J, Rezatofighi H, Yu F, Tao D, Geiger A. Unifying flow, stereo and depth estimation. *IEEE Trans Pattern Anal Mach Intell*. 2023;45:13941–13958. 10.1109/TPAMI.2023.3298645 37490383

[31] Chen F, Fei J, Chen Y, Huang C. Decoupled consistency for semi-supervised medical image segmentation. In: *International Conference on Medical Image Computing and Computer-Assisted Intervention*. Cham (Switzerland): Springer; 2023. p. 551–561.

[32] Wang H, Zhang Q, Li Y, Li X. Allspark: Reborn labeled features from unlabeled in transformer for semi-supervised semantic segmentation. In: *Proceedings of the IEEE/CVF Conference on Computer Vision and Pattern Recognition*. Piscataway (NJ): IEEE; 2024. p. 3627–3636.

[33] Zhu Y, Newsam S. Densenet for dense flow. In: *2017 IEEE International Conference on Image Processing (ICIP)*. Piscataway (NJ): IEEE; 2017. p. 790–794.

[34] He K, Zhang X, Ren S, Sun J. Deep residual learning for image recognition. In: *Proceedings of the IEEE Conference on Computer Vision and Pattern Recognition*. Piscataway (NJ): IEEE; 2016. p. 770–778.

[35] Vaswani A, Shazeer N, Parmar N, et al. Attention is all you need. Adv Neural Inf Proces Syst. 2017;30.

[36] Boyd JH, Forbes J, Nakada TA, Walley KR, Russell JA. Fluid resuscitation in septic shock: A positive fluid balance and elevated central venous pressure are associated with increased mortality. Crit Care Med. 2011;39:259–265. 20975548 10.1097/CCM.0b013e3181feeb15

[37] Leal-Villarreal MA, Aguirre-Villarreal D, Vidal-Mayo JJ, Argaiz ER, Garc´ıa-Ju´arez I. Correlation of internal jugular vein collapsibility with central venous pressure in patients with liver cirrhosis. Off J Am Coll Gastroenterol ACG. 2023;118:1684–1687. 10.14309/ajg.0000000000002315 37146133

[38] Bano S, Qadeer A, Akhtar A, Attaur-Rehman M, Munawar K, Hussain SW, Tariq M, Zafar R. Measurement of internal jugular vein and common carotid artery diameter ratio by ultrasound to estimate central venous pressure. Cureus. 2018;10(3): Article e2277. 30949421 10.7759/cureus.2277 PMC6440552

[39] Ouali Y, Hudelot C, Tami M. Semi-supervised semantic segmentation with cross-consistency training. In: *Proceedings of the IEEE/CVF Conference on Computer Vision and Pattern Recognition*. Piscataway (NJ): IEEE; 2020. p. 12674–12684.

[40] Han K, Sheng VS, Song Y, Liu Y, Qiu C, Ma S, Liu Z. Deep semi-supervised learning for medical image segmentation: A review. Expert Syst Appl. 2024;245: Article 123052.

[41] Krizhevsky A, Sutskever I, Hinton GE. ImageNet classification with deep convolutional neural networks. Adv Neural Inf Proces Syst. 2012;60(6):84–90.

