# Supplementary material for: AI-Enabled Wearable Ultrasound for Noninvasive Central Venous Pressure Monitoring
Source: Cyborg Bionic Syst. 2026 Aug 5;7:0653. doi: 10.34133/cbsystems.0653 (PMC13438027; doi:10.34133/cbsystems.0653)
Supplement: Supplementary file 1 [file cbsystems.0653.f1.zip › Figure S1.pdf]

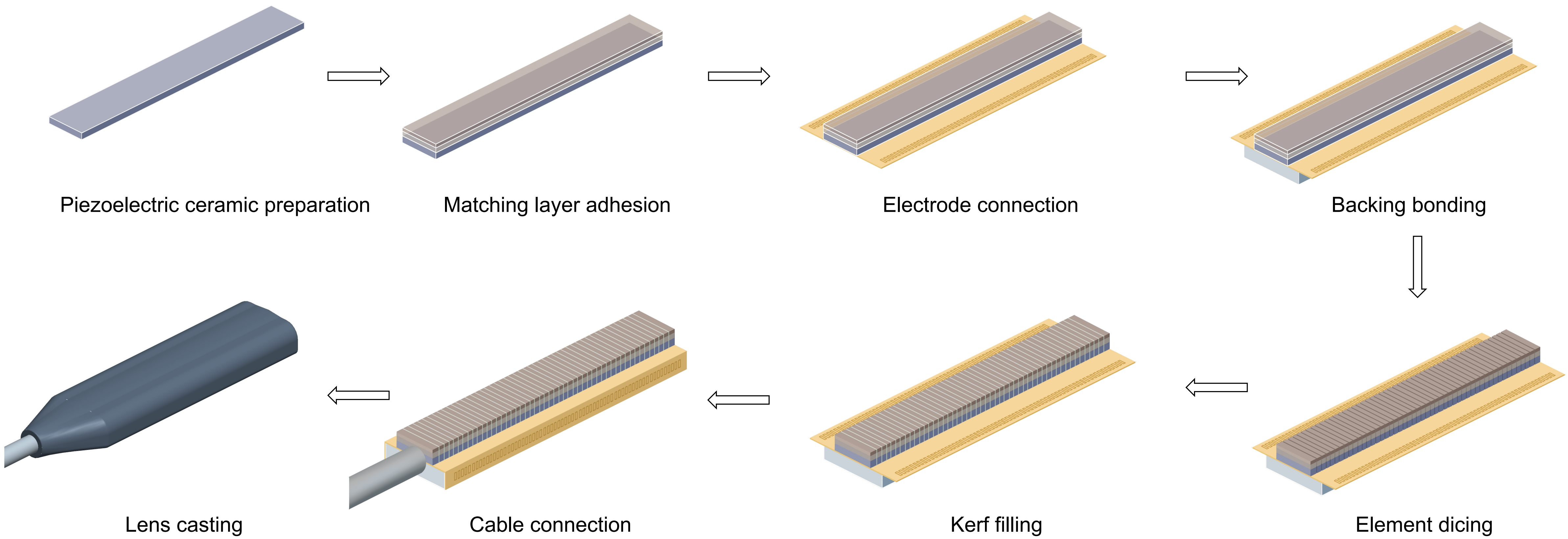
Piezoelectric ceramic preparation
Matching layer adhesion
Electrode connection
Backing bonding
Element dicing
Kerf filling
Cable connection
Lens casting
