# Supplementary material for: AI-Enabled Wearable Ultrasound for Noninvasive Central Venous Pressure Monitoring
Source: Cyborg Bionic Syst. 2026 Aug 5;7:0653. doi: 10.34133/cbsystems.0653 (PMC13438027; doi:10.34133/cbsystems.0653)
Supplement: Supplementary file 1 [file cbsystems.0653.f1.zip › Figure S2.pdf]

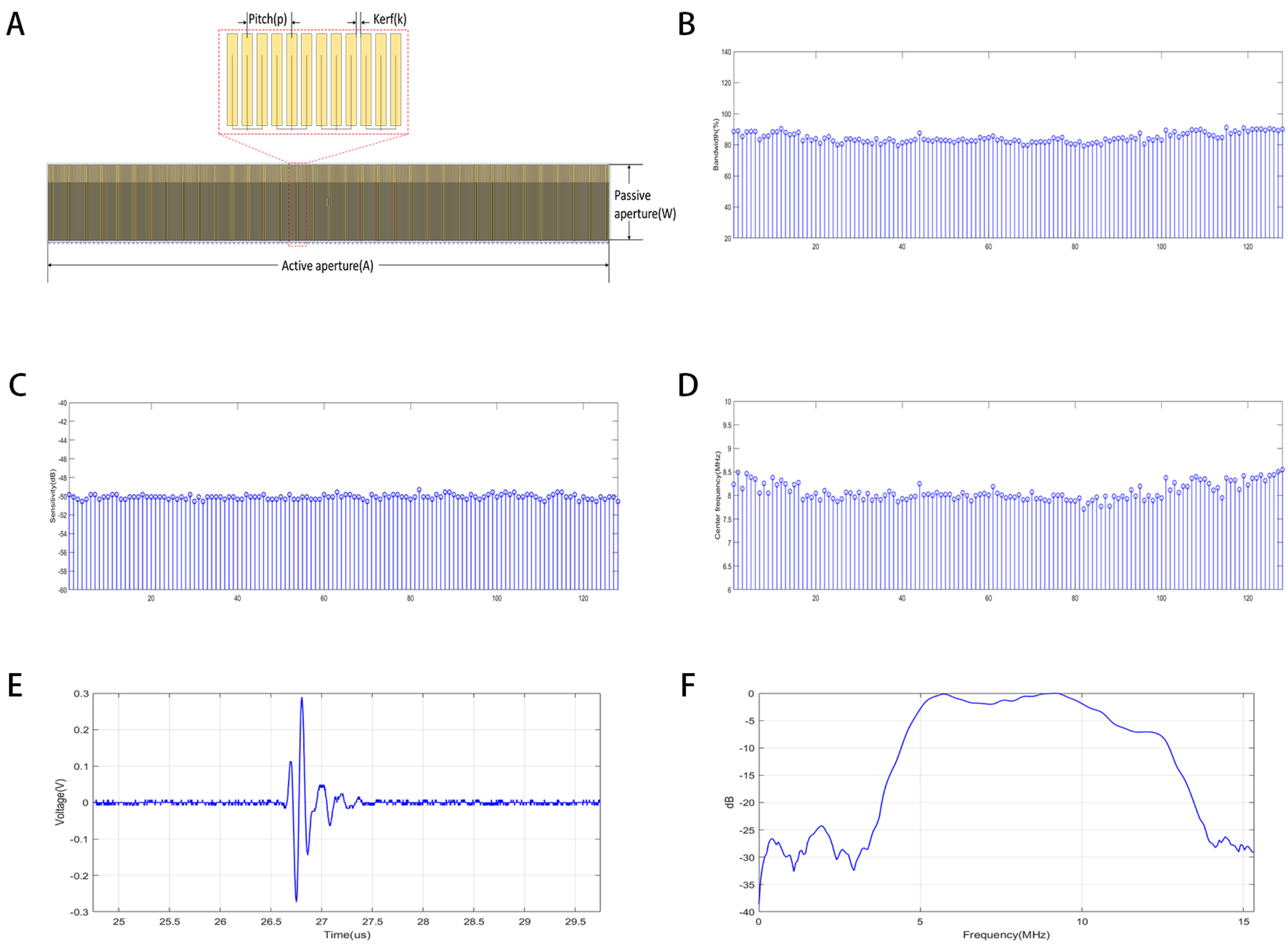
A
Pitch(p)
Kerf(k)
Passive
aperture(W)
Active aperture(A)
B
Bandwidth(%)
C
Sensitivity(dB)
D
Center frequency(MHz)
E
Voltage(V)
Time(us)
F
dB
Frequency(MHz)
