# Supplementary material for: AI-Enabled Wearable Ultrasound for Noninvasive Central Venous Pressure Monitoring
Source: Cyborg Bionic Syst. 2026 Aug 5;7:0653. doi: 10.34133/cbsystems.0653 (PMC13438027; doi:10.34133/cbsystems.0653)
Supplement: Supplementary file 1 [file cbsystems.0653.f1.zip › Figure S3.pdf]

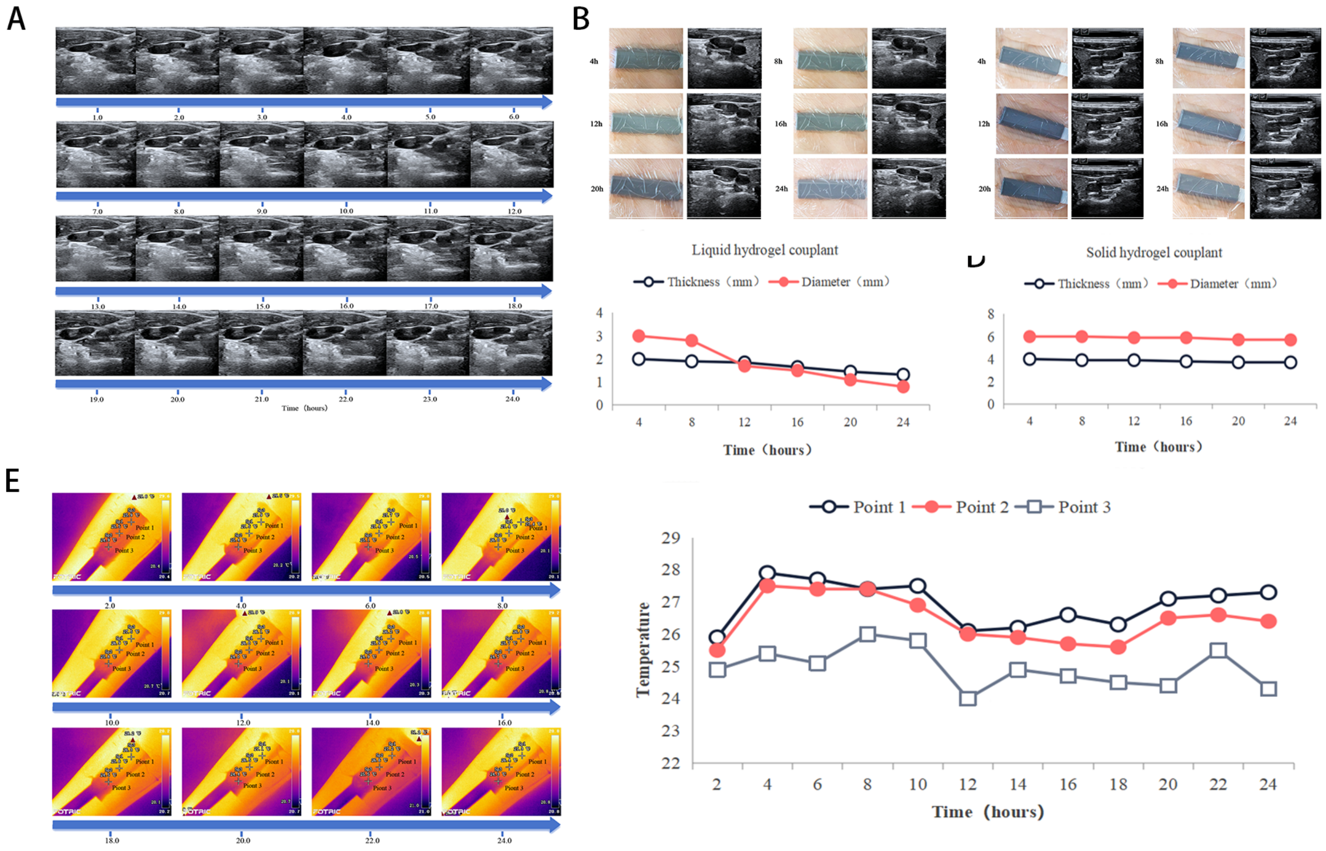
A
B
Liquid hydrogel couplant
Thickness（mm）
Diameter（mm）
Time（hours）
Solid hydrogel couplant
Thickness（mm）
Diameter（mm）
Time（hours）
E
Point 1
Point 2
Point 3
Temperature
Time（hours）
