# Supplementary material for: AI-Enabled Wearable Ultrasound for Noninvasive Central Venous Pressure Monitoring
Source: Cyborg Bionic Syst. 2026 Aug 5;7:0653. doi: 10.34133/cbsystems.0653 (PMC13438027; doi:10.34133/cbsystems.0653)
Supplement: Supplementary file 1 [file cbsystems.0653.f1.zip › Figure S4.pdf]

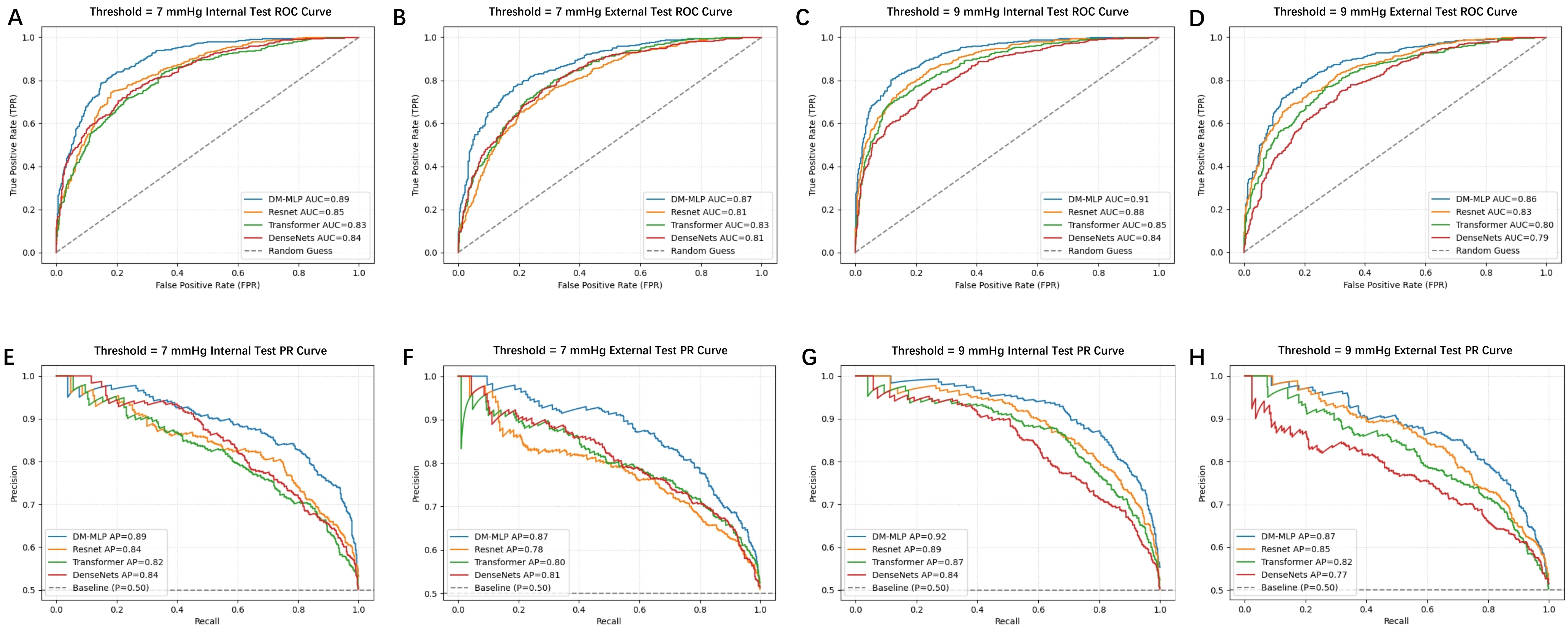

A
Threshold = 7 mmHg Internal Test ROC Curve
True Positive Rate (TPR)
False Positive Rate (FPR)
DM-MLP AUC=0.89
Resnet AUC=0.85
Transformer AUC=0.83
DenseNets AUC=0.84
Random Guess
B
Threshold = 7 mmHg External Test ROC Curve
True Positive Rate (TPR)
False Positive Rate (FPR)
DM-MLP AUC=0.87
Resnet AUC=0.81
Transformer AUC=0.83
DenseNets AUC=0.81
Random Guess
C
Threshold = 9 mmHg Internal Test ROC Curve
True Positive Rate (TPR)
False Positive Rate (FPR)
DM-MLP AUC=0.91
Resnet AUC=0.88
Transformer AUC=0.85
DenseNets AUC=0.84
Random Guess
D
Threshold = 9 mmHg External Test ROC Curve
True Positive Rate (TPR)
False Positive Rate (FPR)
DM-MLP AUC=0.86
Resnet AUC=0.83
Transformer AUC=0.80
DenseNets AUC=0.79
Random Guess
E
Threshold = 7 mmHg Internal Test PR Curve
Precision
Recall
DM-MLP AP=0.89
Resnet AP=0.84
Transformer AP=0.82
DenseNets AP=0.84
Baseline (P=0.50)
F
Threshold = 7 mmHg External Test PR Curve
Precision
Recall
DM-MLP AP=0.87
Resnet AP=0.78
Transformer AP=0.80
DenseNets AP=0.81
Baseline (P=0.50)
G
Threshold = 9 mmHg Internal Test PR Curve
Precision
Recall
DM-MLP AP=0.92
Resnet AP=0.89
Transformer AP=0.87
DenseNets AP=0.84
Baseline (P=0.50)
H
Threshold = 9 mmHg External Test PR Curve
Precision
Recall
DM-MLP AP=0.87
Resnet AP=0.85
Transformer AP=0.82
DenseNets AP=0.77
Baseline (P=0.50)
