# Supplementary material for: AI-Enabled Wearable Ultrasound for Noninvasive Central Venous Pressure Monitoring
Source: Cyborg Bionic Syst. 2026 Aug 5;7:0653. doi: 10.34133/cbsystems.0653 (PMC13438027; doi:10.34133/cbsystems.0653)
Supplement: Supplementary file 1 [file cbsystems.0653.f1.zip › Supplementary Materials.docx]

## Supplementary Materials

### Text S1: Design and fabrication of the Wearable Ultrasound Patch Probe

The fabrication workflow of the wearable ultrasound patch probe is illustrated in Figure S1, including piezoelectric ceramic preparation, matching layer adhesion, electrode connection, backing bonding, element dicing, kerf filling, cable connection and lens casting.

To enhance acoustic energy transmission between the piezoelectric elements and biological tissue, a dual-layer acoustic matching structure was adopted. The acoustic impedances of the two matching layers were 10.5 MRayl and 3 MRayl, with corresponding thicknesses of 0.09 mm and 0.075 mm, respectively. This configuration provides a gradual impedance transition, contributing to bandwidth broadening and improved overall acoustic performance.

A 4-mm-thick backing layer was integrated at the rear of the probe. The thickness was selected based on engineering considerations of operating frequency and material attenuation characteristics. This design suppresses ringing and shortens pulse duration while avoiding significant reduction in output sensitivity, thereby achieving a balance between bandwidth enhancement and transmit efficiency.

The measured bandwidth, center frequency, and pulse-echo response are shown in Figure S1. Detailed design parameters are summarized in Table S1.

### Movies S1:Demonstration of the wearable ultrasound device

The wearable ultrasound device supports various imaging modes, such as 2D, color, and pulsed wave (PW) Doppler. The ultrasound physician captures video images, which are then uploaded imaging data to the hospital server for segmentation using the Dual-decoder spatiotemporal attention network (DSTA-Net). These segmented images, along with basic clinical data, are processed by the Dual-Mixing Multi-Layer Perceptron (DM-MLP) model to predict elevated central venous pressure ($\geq8$ mmHg).

Figure S1: Fabrication workflow of the wearable ultrasound patch probe, including piezoelectric ceramic preparation, matching layer adhesion, electrode connection, backing bonding, element dicing, kerf filling, cable connection and lens casting.

Figure S2: Design schematic and acoustic performance characterization of the 128-element linear array transducer. (A) Schematic illustration of the linear array structure, highlighting key geometric parameters including element pitch (p), kerf (k), active aperture (A), and passive aperture (W). (B–D) Statistical distributions of acoustic parameters across all 128 channels: (B) fractional bandwidth, (C) sensitivity, and (D) center frequency. The relatively small variations across elements indicate good fabrication consistency.(E–F) Representative pulse-echo measurements from a typical element (Channel 73). (E) Time-domain pulse-echo waveform. (F) Corresponding frequency spectrum of the received signal.

Figure S3: Long-term performance stability and thermal safety evaluation of the wearable ultrasound device. (A) Time-lapse ultrasound imaging over a 24-hour period. Representative hourly frames demonstrate stable image quality without visible signal degradation. (B–D) Dehydration resistance comparison of liquid and solid hydrogel couplants. (B) Optical images and corresponding ultrasound scans acquired at 4-hour intervals. The liquid hydrogel (left) exhibits progressive shrinkage and dehydration, whereas the solid hydrogel (right) maintains dimensional stability. (C–D) Quantitative measurements of thickness and diameter over time for the liquid (C) and solid (D) hydrogels. The liquid hydrogel shows noticeable reductions in both thickness and diameter, while the solid hydrogel remains relatively stable. (E–F) Thermal behavior during continuous operation. (E) Infrared thermal images acquired every 2 hours using a FOTRIC 225 camera. (F) Temperature profiles measured at three representative points on the probe. All experiments were conducted at an ambient temperature of 24 C and 30–50% relative humidity. The recorded surface temperatures remained within a physiologically comfortable range (24–28 C), suggesting safe operation during prolonged use.

Figure S4: Sensitivity analysis of model performance under alternative central venous pressure (CVP) thresholds. (A–D) Receiver operating characteristic (ROC) curves of DM-MLP, ResNet, Transformer, and DenseNet evaluated at CVP thresholds of 7 mmHg and 9 mmHg in the internal and external test sets. (A, B) Threshold = 7 mmHg; (C, D) Threshold = 9 mmHg. Internal test set: (A, C); External test set: (B, D). (E–H) Precision–recall (PR) curves under the same threshold settings. (E, F) Threshold = 7 mmHg; (G, H) Threshold = 9 mmHg. Internal test set: (E, G); External test set: (F, H).

Table S1: Detailed design parameters and acoustic specifications of the wearable patch probe

| **Parameter** | **Value** |
| --- | --- |
| Center Frequency (MHz) | 8.50 |
| Probe Type | Linear array |
| Number of channels | 128 |
| Element pitch (mm) | 0.30 |
| Kerf (mm) | 0.028 |
| Active aperture (mm) | 38.40 |
| Passive aperture (mm) | 4.20 |
| Focal depth (mm) | 30 |
| Cable length (cm) | 120 |
| Match layer 1 acousticimpedance Zm (MRayl) | 10.50 |
| Match layer 2 acousticimpedance Zm (MRayl) | 3 |
| Match layer 1 thickness (mm) | 0.09 |
| Match layer 2 thickness (mm) | 0.08 |
| Backing layer thickness (mm) | 4 |

Table S2: Cross-center cohort composition and key demographic differences

| **Dataset** | **Center** | **N total** | **Elevated CVP n (%)** | **Age (Elevated CVP) mean**$\pm$**SD** | **Female (Elevated CVP) n (%)** | **Age (Non-elevated CVP) mean**$\pm$**SD** | **Female (Non-elevated CVP) n (%)** |
| --- | --- | --- | --- | --- | --- | --- | --- |
| Training set | Shanghai Sixth Hospital | 218 | 87 (39.9%) | 58.86$\pm$ 18.14 | 38 (43.7%) | 52.21$\pm$ 21.27 | 77 (58.8%) |
| Internal test set | Shanghai Sixth Hospital | 54 | 22 (40.7%) | 58.05$\pm$ 16.03 | 12 (54.5%) | 52.47$\pm$ 18.10 | 17 (53.1%) |
| External test set | Shanghai Tenth Hospital | 77 | 27 (35.1%) | 58.96$\pm$ 17.22 | 12 (44.4%) | 50.98$\pm$ 16.73 | 22 (44.0%) |

Elevated CVP was defined as CVP $\geq$ 8 mmHg. Data are presented as mean$\pm$ SD or n (%). CVP, central venous pressure; SD, standard deviation.

Table S3: Inter- and intra-observer reliability of IJV and CCA measurements

!

|  |  | **IJV Max Area** (mm) | **IJV Min Area** (mm) | **IJV Ratio** | **CCA Area** (mm) | **IJV Max/CCA Area** |
| --- | --- | --- | --- | --- | --- | --- |
| 3***Inter-observer agreement** | **ICC** | 0.997 | 0.999 | 0.997 | 0.994 | 0.999 |
|  | **95% CI** | 0.993$-$ 0.999 | 0.998$-$ 1.000 | 0.992$-$ 0.999 | 0.984$-$ 0.997 | 0.998$-$ 1.000 |
|  | ***P*** | $<0.001$ | | | | |
| 3***Intra-observer agreement** | **ICC** | 0.998 | 0.999 | 0.996 | 0.994 | 0.998 |
|  | **95% CI** | 0.995$-$ 0.999 | 0.998$-$ 1.000 | 0.989$-$ 0.998 | 0.985$-$ 0.998 | 0.995$-$ 0.999 |
|  | ***P*** | $<0.001$ | | | | |

Note. IJV Max Area, internal jugular vein maximum area; IJV Min Area, internal jugular vein minimum area; IJV Ratio = (IJV maximum area - IJV minimum area)/IJV maximum area; CCA Area, common carotid artery area at end-expiration; IJV Max/CCA Area, ratio of IJV maximum area to CCA area at end-expiration; ICC, intraclass correlation coefficient; 95% CI, 95% confidence interval.

Table S4: The B-A consistency analysis between expert manual and DSTA-Net model

|  | Internal test set (54 patients) | | External test set (77 patients) | |
| --- | --- | --- | --- | --- |
|  | Mean Difference | LOA | Mean Difference | LOA |
| IJV Max Area (% error) | 11.68 | (2.58, 20.7) | 1.31 | (-21.65, 24.26) |
| IJV Min Area (% error) | 4.93 | (3.21, 6.65) | -0.68 | (-16.06, 14.69) |
| IJV Ratio (% error) | 21.73 | (-50.14, 93.60) | 8.04 | (-75.41, 91.48) |
| CCA Area (% error) | 9.59 | (3.14, 16.04) | -1.54 | (-10.96, 7.88) |
| IJV Max /CCA (% error) | 2.10 | (-8.05, 12.25) | 2.84 | (-21.33, 27.01) |

IJV Max Area, internal jugular vein maximal area; IJV Min Area, internal jugular vein minimal area; IJV Ratio = (IJV maximum area-IJV minimum area)/IJV maximum area; CCA Area, common carotid artery area at end-expiration; IJV Max Area/CCA Area, ratio of IJV maximal area to CCA area at end-expiration; Mean difference represents the average percentage error between the DSTA-Net model and expert manual measurements, while LOA (limits of agreement) indicates the range within which 95% of the measurement differences are expected to fall. The LOA is expressed as the mean difference $\pm$ 1.96 standard deviations, providing a measure of the agreement or variability in error between the two methods.

Table S5: ROC Curve Performance Metrics of DM-MLP Model vs. Baseline Models on Internal Test Set

|  | **P** | **AUC** | **Accuracy** | **Sensitivity** | **Specificity** | **PPV** | **NPV** |
| --- | --- | --- | --- | --- | --- | --- | --- |
| **DM-MLP** | 4***<0.05** | **0.91[0.89,0.93]** | **0.833**$\pm$ 0.028 | **0.800**$\pm$ 0.022 | **0.875**$\pm$ 0.025 | **0.889**$\pm$ 0.021 | **0.778**$\pm$ 0.048 |
| **ResNet** |  | **0.87[0.85,0.89]** | 0.796$\pm$ 0.025 | 0.767$\pm$ 0.046 | 0.833$\pm$ 0.021 | 0.852$\pm$ 0.016 | 0.741$\pm$ 0.033 |
| **Transformer** |  | **0.84[0.81,0.87]** | 0.796$\pm$ 0.032 | 0.733$\pm$ 0.050 | 0.875$\pm$ 0.030 | 0.880$\pm$ 0.024 | 0.724$\pm$ 0.040 |
| **DenseNets** |  | **0.85[0.83,0.87]** | 0.796$\pm$ 0.029 | 0.700$\pm$ 0.035 | 0.917$\pm$ 0.018 | 0.913$\pm$ 0.019 | 0.710$\pm$ 0.031 |

Data in brackets are 95% CIs. The p corresponds to the statistical significance test performed on the calculated AUC. AUC, Area under the receiver operating characteristic curve; NPV, Negative predictive value; PPV, Positive predictive value.

Table S6: ROC Curve Performance Metrics of DM-MLP Model vs. Baseline Models on External Test Set

|  | **P** | **AUC** | **Accuracy** | **Sensitivity** | **Specificity** | **PPV** | **NPV** |
| --- | --- | --- | --- | --- | --- | --- | --- |
| **DM-MLP** | 4***<0.05** | **0.87[0.85,0.89]** | **0.818**$\pm$ 0.028 | **0.880**$\pm$ 0.022 | **0.704**$\pm$ 0.042 | **0.846**$\pm$ 0.021 | **0.760**$\pm$ 0.048 |
| **ResNet** |  | **0.82[0.79,0.85]** | 0.779$\pm$ 0.025 | 0.840$\pm$ 0.027 | 0.667$\pm$ 0.052 | 0.824$\pm$ 0.016 | 0.692$\pm$ 0.047 |
| **Transformer** |  | **0.81[0.78,0.84]** | 0.740$\pm$ 0.032 | 0.800$\pm$ 0.030 | 0.630$\pm$ 0.069 | 0.800$\pm$ 0.024 | 0.630$\pm$ 0.056 |
| **DenseNets** |  | **0.79[0.76,0.82]** | 0.753$\pm$ 0.029 | 0.800$\pm$ 0.020 | 0.667$\pm$ 0.066 | 0.816$\pm$ 0.029 | 0.643$\pm$ 0.044 |

Data in brackets are 95% CIs. The p corresponds to the statistical significance test performed on the calculated AUC. AUC, Area under the receiver operating characteristic curve; NPV, Negative predictive value; PPV, Positive predictive value.

Table S7: Abbreviations and Full Forms

| **Abbreviation** | **Full Form** |
| --- | --- |
| AI | Artificial intelligence |
| Arr-Mix | Attribute-Mixing |
| AUC | Area under the curve |
| AUC–PR | Precision–recall curve |
| AUC–ROC | Receiver operating characteristic curve |
| B-A analysis | Bland–Altman analysis |
| BCE | Binary cross-entropy |
| BMI | Body mass index |
| BSA | Body surface area |
| Case-Mix | Case-Mixing |
| CCA | Common carotid artery |
| CCA Area | The CCA area at end-expiration |
| CIs | Confidence intervals |
| CVC | Central venous catheter |
| CVP | Central venous pressure |
| DBP | Diastolic blood Pressure |
| Dice | Dice coefficient |
| DMBs | Dual mixing blocks |
| DM-MLP | Dual-modality multilayer perceptron |
| DSTA-Net | Dual-decoder spatiotemporal attention network |
| FFN | Fee forward network |
| GT | Ground truth |
| HR | Heart rate |
| ICUs | Intensive care units |
| IJV | Internal jugular vein |
| IJV Max Area | The maximum IJV area at end-expiration |
| IJV Min Area | The minimum IJV area at end-inspiration |
| IJV Max/CCA Area | The maximum IJV area /CCA area |
| IJV Ratio | The change rate of the IJV |
| IoU | Intersection over Union |
| IQRs | Interquar-tile ranges |
| LOA | Limits of agreement |
| NPV | Negative predictive value |
| PPV | Positive predictive value |
| PW | Pulsed wave |
| PZT-5H | Lead Zirconate Titanate-5H |
| ROC | Receiver operating characteristic |
| SBP | Systolic blood Pressure |
| SD | Standard deviation |
